# Anthrax edema toxin disrupts distinct steps in Rab11-dependent junctional transport

**DOI:** 10.1371/journal.ppat.1006603

**Published:** 2017-09-25

**Authors:** Annabel Guichard, Prashant Jain, Mahtab Moayeri, Ruth Schwartz, Stephen Chin, Lin Zhu, Beatriz Cruz-Moreno, Janet Z. Liu, Bernice Aguilar, Andrew Hollands, Stephen H. Leppla, Victor Nizet, Ethan Bier

**Affiliations:** 1 Section of Cell and Developmental Biology, University of California, San Diego, La Jolla, CA, United States of America; 2 Laboratory of Parasitic Diseases, National Institute of Allergy and Infectious Diseases, NIH, Bethesda, MD, United States of America; 3 Department of Pediatrics, University of California, San Diego, La Jolla, CA, United States of America; 4 Division of Pediatric Infectious Diseases and the Immunobiology Research Institute, Cedars-Sinai Medical Center, Los Angeles, CA 90048, United States of America; 5 Skaggs School of Pharmacy & Pharmaceutical Sciences, University of California, San Diego, La Jolla, CA, United States of America; University of Illinois, UNITED STATES

## Abstract

Various bacterial toxins circumvent host defenses through overproduction of cAMP. In a previous study, we showed that edema factor (EF), an adenylate cyclase from *Bacillus anthracis*, disrupts endocytic recycling mediated by the small GTPase Rab11. As a result, cargo proteins such as cadherins fail to reach inter-cellular junctions. In the present study, we provide further mechanistic dissection of Rab11 inhibition by EF using a combination of *Drosophila* and mammalian systems. EF blocks Rab11 trafficking after the GTP-loading step, preventing a constitutively active form of Rab11 from delivering cargo vesicles to the plasma membrane. Both of the primary cAMP effector pathways -PKA and Epac/Rap1- contribute to inhibition of Rab11-mediated trafficking, but act at distinct steps of the delivery process. PKA acts early, preventing Rab11 from associating with its effectors Rip11 and Sec15. In contrast, Epac functions subsequently via the small GTPase Rap1 to block fusion of recycling endosomes with the plasma membrane, and appears to be the primary effector of EF toxicity in this process. Similarly, experiments conducted in mammalian systems reveal that Epac, but not PKA, mediates the activity of EF both in cell culture and *in vivo*. The small GTPase Arf6, which initiates endocytic retrieval of cell adhesion components, also contributes to junctional homeostasis by counteracting Rab11-dependent delivery of cargo proteins at sites of cell-cell contact. These studies have potentially significant practical implications, since chemical inhibition of either Arf6 or Epac blocks the effect of EF in cell culture and *in vivo*, opening new potential therapeutic avenues for treating symptoms caused by cAMP-inducing toxins or related barrier-disrupting pathologies.

## Author summary

Recent anthrax outbreaks in Zambia and northern Russia and biodefense preparedness highlight the need for new therapies to counteract fatal late-stage pathologies in patients infected with *Bacillus anthracis*. Indeed, two toxins secreted by this pathogen—edema toxin (ET) and lethal toxin (LT)—can cause death in face of effective antibiotic treatment. ET, a potent adenylate cyclase, severely impacts host cells and tissues through an overproduction of the ubiquitous second messenger cAMP. Previously, we identified Rab11 as a key host factor inhibited by ET. Blockade of Rab11-dependent endocytic recycling resulted in the disruption of intercellular junctions, likely contributing to life threatening vascular effusion observed in anthrax patients. Here we present a multi-system analysis of the mechanism by which EF inhibits Rab11 and exocyst-dependent trafficking. Epistasis experiments in *Drosophila* reveal that over-activation of the cAMP effectors PKA and Epac/Rap1 interferes with Rab11-mediated trafficking at two distinct steps. We further describe conserved roles of Epac and the small GTPase Arf6 in ET-mediated disruption of vesicular trafficking and show how chemical inhibition of either pathway greatly alleviates ET-induced edema. Thus, our study defines Epac and Arf6 as promising drug targets for the treatment of infectious diseases and other pathologies involving cAMP overload or related barrier disruption.

## Introduction

Bacterial pathogens enhance infectivity by secreting toxins that deregulate immune signaling pathways or disrupt host cellular barriers. One class of toxins produced by diverse bacterial species dramatically increases intracellular concentrations of cAMP. This striking evolutionary convergence suggests that over-production of this second messenger represents a successful strategy to promote growth and dissemination of infectious agents and associated disease symptoms [1]. These toxins include adenylate cyclases (AC), such as edema factor (EF) from *Bacillus anthracis* (*B*. *a*.), CyaA from *Bordetella pertussis*, and ExoY from *Pseudomonas aeruginosa*. Other toxins modify host proteins to induce cAMP production by endogenous cellular machineries. For example, cholera toxin (Ctx) from *Vibrio cholerae*, and the related heat-labile toxin from *enterotoxigenic Escherichia coli*, both ADP-ribosylate the α subunit of trimeric G proteins to stimulate cAMP synthesis by host AC, while pertussis toxin (Ptx) from *Bordetella pertussis* ADP-ribosylates and inactivates Gi subunits that normally inhibit endogenous ACs (reviewed in [2]).

*B*.*a*., the etiological agent of anthrax, produces two A-subunit toxins, edema factor (EF) and lethal factor (LF), which are secreted together with a shared B-subunit, protective antigen (PA), and then assemble to form edema toxin (ET) and lethal toxin (LT), respectively [3,4]. ET and LT can enter a wide array of mammalian cells expressing either of two related surface receptors, CMG2 or TEM8, where upon the toxins are internalized, leading to the release of the enzymatic A-subunits into the cytoplasm [5]. LF is a zinc metalloprotease that cleaves and inactivates mitogen-activated protein kinase kinases (MAPKKs or MEKs) to block MAPK signaling pathways [6] and, in some hosts, also cleaves NLRP1 to activate the inflammasome [7]. EF is a calmodulin-dependent AC, estimated to be more than a hundred times more potent than its mammalian counterparts in raising intracellular cAMP concentrations [8]. During the early stages of anthrax infection, LT and ET inhibit the innate immune response, reducing cell viability, disrupting chemotaxis and phagocytosis and deregulating cytokine production by macrophages, dendritic cells, and lymphocytes. These combined toxic effects promote bacterial growth and dissemination throughout the host [9,10]. In late fulminant stages of the disease, increasing amounts of ET [11] are released into the bloodstream, and in combination with LT cause edema, bleeding and hemorrhagic lesions (ET), and atypical collapse of the cardiovascular system (LT), often culminating in cardiac arrest and death [12,13].

Molecular pathways altered by the concerted effects of EF and LF were analyzed in transgenic *Drosophila* models by tissue-specific and conditional expression of the A-toxin subunit using the GAL4/UAS system [14]. Expression in the developing wing revealed that EF caused a phenotype very similar to that of a dominant-negative form of Rab11, a small GTPase of the Rab subfamily essential for endocytic recycling [15,16]. Consistent with EF blocking Rab11-dependent trafficking, two known cargo proteins, Delta (a transmembrane ligand activating the Notch receptor) and the homophylic adhesion protein E-cadherin[17,18] failed to reach their normal destination at apical adherens junctions (AJs). In addition, Rab11 levels were severely reduced in response to EF expression in the wing imaginal disc. This newly recognized activity of EF was also observed in mammalian cells, where ET caused a clear disruption of AJs and Notch signaling in several endothelial cell lines, and was essential for *B*. *a*.-induced vascular effusion *in vivo* [19]. To promote cargo vesicle fusion with the plasma membrane at proper apical sites, Rab11 relies on its effector Sec15, which physically binds to the GTP-bound/active form of Rab11[13,20,21]. Sec15 is a key component of the exocyst, an octameric protein complex that triggers docking and SNARE-mediated fusion of cargo vesicles with the plasma membrane [22]. When over-expressed in various cell types, Sec15 promotes the assembly of large punctate structures[20] that also contain Rab11, Sec15, and other exocyst components. Consistent with previous observations, we found that EF prevented the formation of such Sec15-rich punctae. Interestingly, LF led to a similar inhibition of Sec15 punctae assembly, although via a Rab11-independent mechanism, indicating that Sec15 acts as a convergence point that integrates the effects of both anthrax toxins to block exocyst-mediated trafficking and disrupt integrity of the endothelial barrier [19].

Subsequent studies revealed that cholera toxin also blocks Rab11-mediated trafficking, an activity expected to increase intestinal epithelial permeability, paracellular water loss and diarrhea [23]. These similar cellular effects of ET and Ctx are likely to contribute to the hallmark pathological features and symptoms associated with anthrax and cholera respectively [24].

In the present studies, we delve deeper into the molecular pathways connecting ET-induced cAMP overload to inhibition of Rab11. We apply a combination of approaches involving GTPase isoform-specific transgenes and antibodies, different *Drosophila* epithelial tissues, human cell lines, and *in vivo* experiments in mice. Our results indicate that EF disrupts Rab11-dependent processes after the GTP loading step. In flies, both cAMP effectors PKA and Epac disrupt Rab11-mediated junctional transport when artificially activated, but disable early versus late steps of the trafficking process, respectively. However, the Epac/Rap1 pathway seems to serve as the primary mediator of EF-induced toxemia in mammalian systems as well as in the *Drosophila* wing epithelium. Constitutive activation of Arf6, a small GTPases involved in endocytic retrieval of junctional proteins [25], causes phenotypes nearly identical to that of EF, and similarly alters Rab11 levels and distribution. These findings have potentially important practical implications, since chemical inhibition of Epac (using the selective cAMP analog ESI-09[26]) or Arf6 (using SecinH3[27] or Slit[28]) can reverse the effect of EF in a mouse footpad edema assay and in human cells. Such small molecule interventions open new potential therapeutic avenues for alleviating pathological effects of cAMP toxins and potentially other barrier disruptive agents.

## Results

### EF blocks Rab11 activity following GTP loading

**Fig 1:**
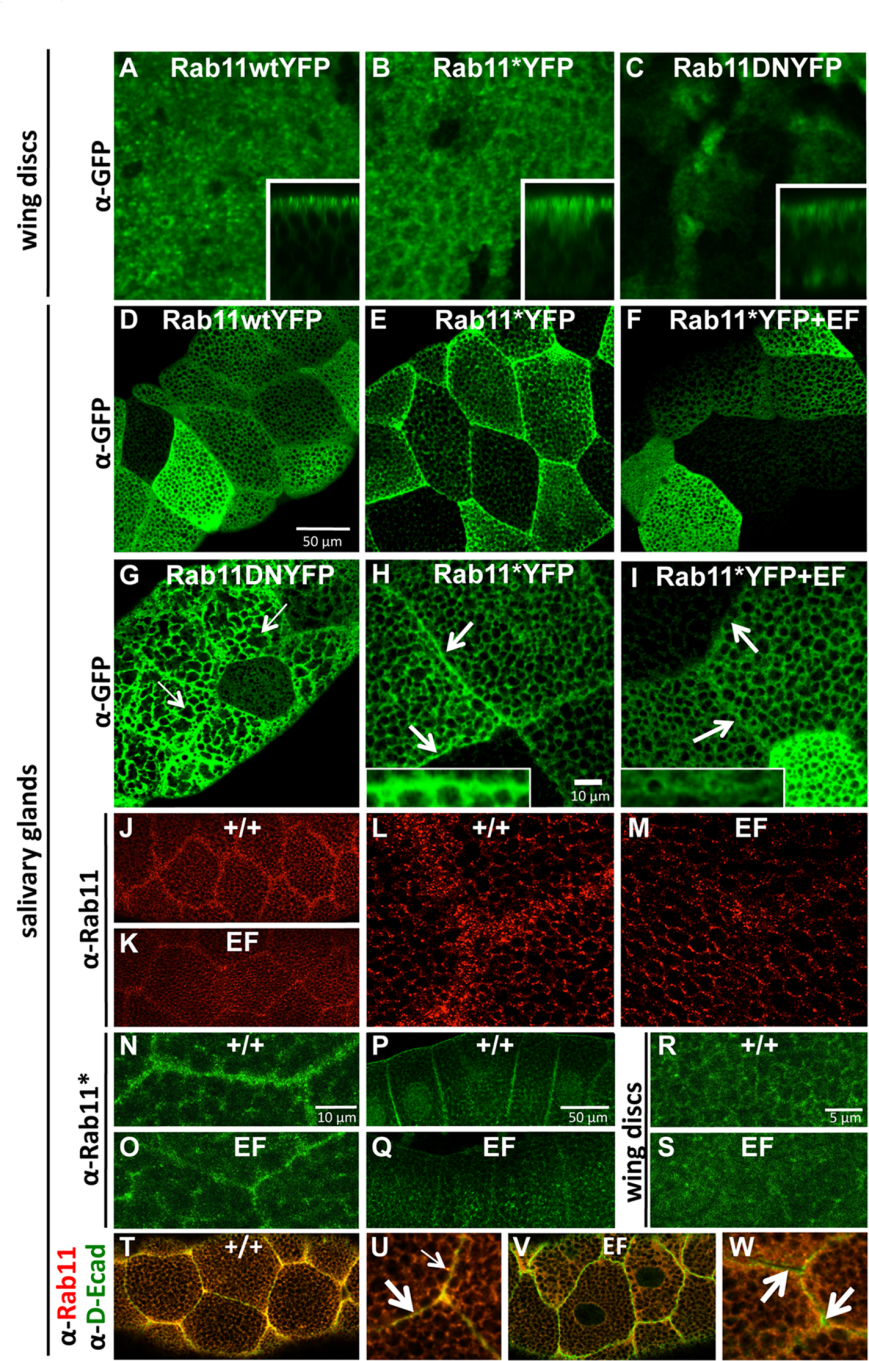
EF inhibits Rab11 downstream of GTP loading. (A-C) Wing imaginal discs expressing wild-type (wt) and mutant forms of YFP-tagged Rab11, using the strong wing-specific MS1096 GAL4 (abbreviated as 1096GAL4), and stained with a Rabbit anti-GFP antibody, reveal different sub-cellular distributions of Rab11. Insets show corresponding Z-sections. (A) Rab11wt, showing apical restriction. (B) Rab11 activated (or Rab11*), showing apical restriction plus junctional concentration, (C) Rab11 Dominant-Negative (or Rab11DN) showing loss of apical/junctional staining. The bipartite staining of Rab11* is even more pronounced in salivary glands (D-I). (D) 1096GAL4>Rab11wtYFP. (E) 1096GAL4>Rab11*YFP with higher magnification in (H). Arrows point at intercellular junctions where Rab11* accumulates. (F) 1096GAL4>Rab11*YFP +EF. EF blocks Rab11* targeting to the junctions, higher magnification shown in (I). Arrows point to intercellular junctions where Rab11* no longer accumulates when co-expressed with EF. (G) 1096GAL4>Rab11DNYFP, showing that Rab11DN does not concentrate at the junctions, but alters the morphology of secretory granules (thin arrows). (J-M): detection of the endogenous Rab11 reveals that it collects near junctions in a punctate pattern (J), with higher magnification in (L). This preference is abrogated in EF-expressing glands (K), with higher magnification in (M). Selective staining of the activated component of endogenous Rab11 (Rab11*) reveals that this functionally relevant form accumulates next to cell junctions in salivary glands (N), sagittal view in (P), an effect that is reduced in EF-expressing glands (O), sagittal view in (Q). The effect of EF on endogenous Rab11* is also visible in wing imaginal discs: (R) wt, (S) 1096GAL4>EF. Co-labeling of Rab11 and D-Ecad, the *Drosophila* ortholog of E-Cadherin (T-W) reveals co-localization of Rab11 and D-Ecad at the AJs in wt salivary glands (T), (U) higher magnification. Thick arrow indicates AJs, thin arrow indicates punctate stain near the AJs. Co-localization is lost upon EF expression (V) in salivary glands, (W) higher magnification.

In an effort better understand how EF blocks Rab11-dependent trafficking, we initially examined the behaviors of three YFP-tagged forms of Rab11: wild-type (wt), activated (*), and dominant-negative (DN) [29]. These variants were first expressed in the wing primordium in which inhibition of Rab11 by EF was initially discovered and analyzed [19,23]. The sub-cellular distribution of Rab11wtYFP detected by immuno-fluorescence appears as a grainy stain restricted primarily to the apical pole of epithelial cells (Fig 1A). In addition to this wt pattern, activated Rab11 (Rab11*YFP), a mutant that cannot hydrolyze GTP to GDP, displayed an additional staining component that accumulates at or near apical adherens junctions (AJs) (Fig 1B). This latter staining is in line with the known role of Rab11 in junctional delivery (see S1 Fig for co-localization of Rab11* and *Drosophila* E-cadherin, D-Ecad). We conclude that active GTP-bound Rab11 is selectively directed to cell-cell contacts at AJs. Consistent with this hypothesis, a dominant-negative Rab11 (Rab11DNYFP), locked in its inactive GDP-bound conformation, did not display a preferential junctional distribution nor apical accumulation (Fig 1C). We then turned our analysis to larval salivary glands, which are comprised of large polyploid secretory cells[30], where the junctional-specific distribution of Rab11*YFP appears more pronounced (Fig 1E). In these cells, over-expressed Rab11wtYFP was distributed throughout the cytoplasm, albeit excluded from densely packed secretory granules, with higher levels detected in the vicinity of intercellular junctions (Fig 1D). Rab11*YFP behaved similarly but, in addition, exhibited a strong junctional staining component (Fig 1E and 1H). In contrast, Rab11DNYFP did not concentrate at junctions, but altered the size and shape of secretory granules (Fig 1G, thin arrows), suggesting that Rab11 normally plays a role in the formation or trafficking of these granules. These findings in the salivary gland confirm our results in wing discs suggesting that the activated GTP-bound form of Rab11 is selectively directed to AJs.

According to the hypothesis that only the activated form of Rab11 traffics to junctions, factors blocking Rab11 upstream of the GTP-loading step should have no effect on Rab11* distribution, whereas inhibitory factors acting downstream of Rab11 should prevent Rab11* from accumulating at AJs. To test this model, we employed two RNAi constructs, one for knocking-down expression of Crag, which is the only known GEF specifically dedicated to activating Rab11 [31], and the other for knocking-down Sec15, an important Rab11 effector required for junctional delivery [18]. Specific inhibitory activities of these RNAi lines were confirmed using epitope-tagged forms of Crag and Sec15 (See S2 Fig). When co-expressed with Rab11*YFP, Sec15-RNAi clearly prevented Rab11*YFP from reaching the AJs (S3 Fig), consistent with Sec15 acting downstream of Rab11 activation. In contrast, Crag-RNAi had no effect on Rab11*YFP distribution, consistent with Crag acting upstream of Rab11 (S3 Fig). Next, we examined whether EF blocks activation (GTP loading) of Rab11 or a subsequent step, by testing the effect of EF on Rab11*YFP localization. Expression of EF blocked all Rab11* junctional accumulation (Fig 1F, compare panels 1H and 1I showing higher magnifications, see S4 Fig for quantifications of junctional Rab11* in response to EF expression). As the constitutively activated mutant Rab11*YFP remains sensitive to EF, we conclude that this toxin acts after the GTP-loading step.

We next examined the behavior of endogenous Rab11 in salivary glands and its response to EF challenge using an antibody that detects all forms of Rab11 (α-Rab11). In wt glands, Rab11 shows a granular distribution with a higher concentration in the vicinity of cell junctions (Fig 1J and 1L), which may represent an enrichment in activated Rab11. In EF-expressing glands, this juxta-junctional staining was clearly reduced: Rab11 dots were detected at similar levels as in wt glands, but very few accumulated around the junctions (Fig 1K and 1M). These findings are consistent with the hypothesis that EF prevents activated Rab11 from reaching the AJs. To test this model further we employed an antibody that specifically detects the activated Rab11 pool (α-Rab11*). Consistent with the observations described above, we found that endogenous activated Rab11 localized predominantly to AJs (Fig 1N and 1P). In EF-expressing glands, the overall levels of activated Rab11* were not obviously altered, however, less activated Rab11 accumulated at the AJs (Fig 1O and 1Q, compare with 1N and 1P). Similarly, in EF-expressing discs, activated Rab11 levels remained comparable to wt levels, while junctional accumulation was severely reduced by EF (Fig 1R and 1S). We conclude that EF does not interfere with Rab11 activation (GTP loading), but instead blocks Rab11 function at a subsequent step(s) to prevent the activated form of Rab11 from trafficking to AJs.

Next, we tested whether the association between Rab11 and its known cargo protein D-Ecad was affected by EF. As expected, co-labeling of Rab11 and D-Ecad in wt salivary glands revealed strong co-localization at cell junctions (Fig 1T and 1U -wide arrow-) and in punctate structures near the junctions (Fig 1U -thin arrow-). In glands expressing EF, however, D-Ecad approached the cell surface (Fig 1V and 1W), but failed to fully localize to AJs, as revealed by gaps in staining between cells (Fig 1W, arrows). Similarly, expression of Rab11DN led to an accumulation of D-Ecad just under the junctions, while many gaps were visible between cells (S5 Fig). These observations suggest that in salivary glands, Rab11 is not required for trafficking D-Ecad to the proximity of junctions, but is critical for the final delivery at the plasma membrane through vesicular fusion. Importantly, in EF-expressing glands, Rab11-DEcad co-localization was abrogated (Fig 1V and 1W, wide arrows). We conclude that EF blocks the association between Rab11 and trafficking vesicles containing cargo proteins such as D-Ecad, leading to a failure in final step of junctional delivery with the consequence of weakened AJs.

### PKA and Epac/Rap1 block Rab11 trafficking at distinct steps

**Fig 2:**
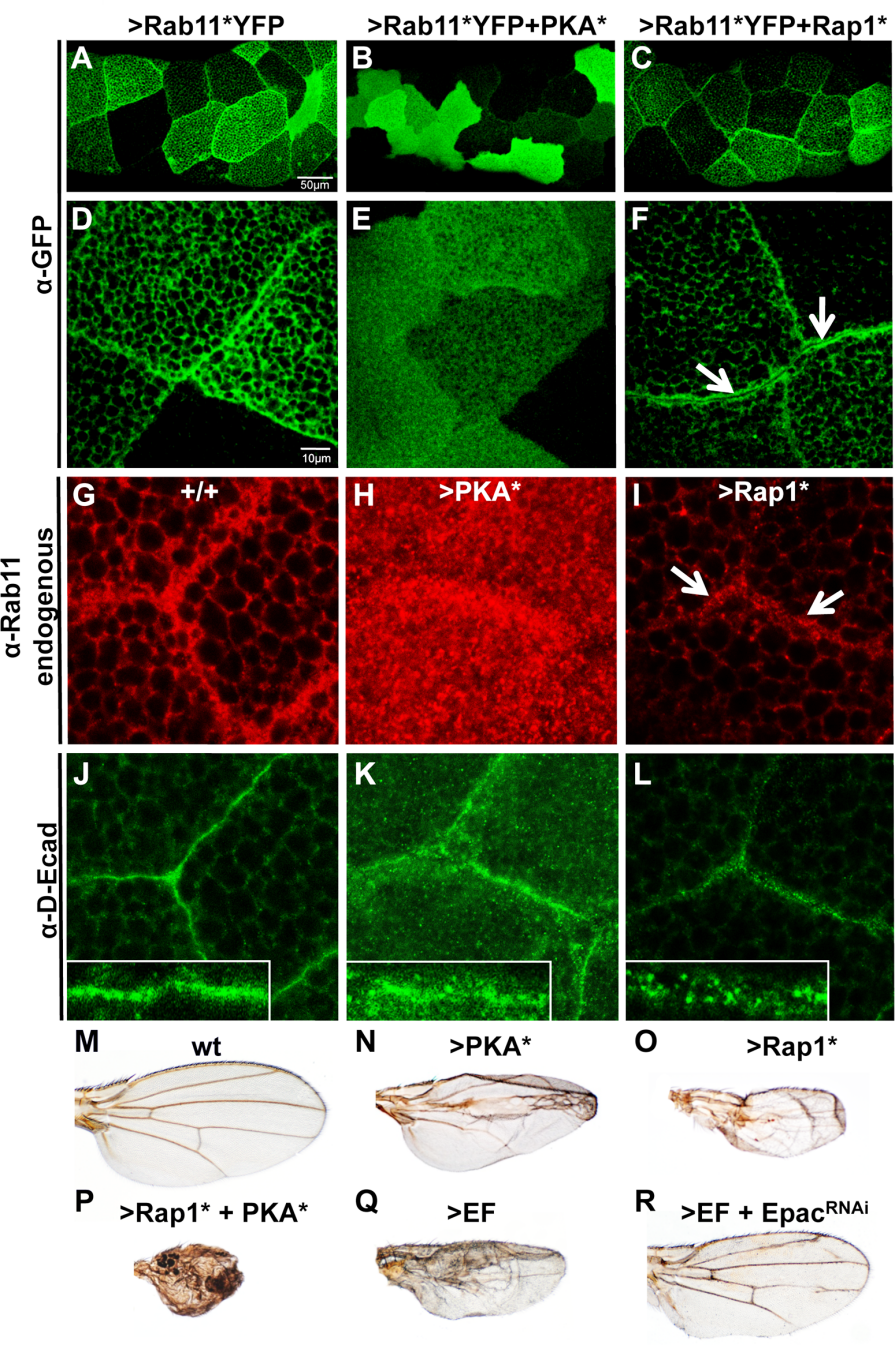
PKA* and Rap1* block Rab11 at distinct steps of junctional trafficking. (A-F) Rab11*YFP distribution (detected by a Rabbit anti-GFP antibody) is differentially affected by PKA* versus Rap1* in *Drosophila* salivary glands. (A) Rab11* (in 1096GAL4>Rab11*YFP glands) shows a strong preference for the intercellular junctions (see D for higher magnification). (B) PKA* induces ubiquitous redistribution of Rab11* (in 1096GAL4>Rab11*YFP+PKA* glands, see E for higher magnification). (C) Rap1* expression does not alter Rab11* targeting to the junctions, but blocks the final membrane fusion event (in 1096GAL4>Rab11*YFP+Rap1* glands, see F for higher magnification). (G-I) Endogenous Rab11 (detected by a mouse anti-Rab11 antibody) in salivary glands of the indicated genotypes (G) Wild-type (+/+). (H) 1096GAL4>PKA*. Rab11 shows higher levels and loss of junctional preference. (I) 1096GAL4>Rap1*, Rab11 still accumulates near the junctions. (J-L) D-Ecad staining of salivary glands. The salivary gland-specific SglGAL4 was used for these stains. (J) Linear AJs form in wild-type glands. (K) PKA* expression results in weakened junctions and cytoplasmic retention of D-ECad in punctate vesicles. (L) Rap1* expression causes D-Ecad to accumulate around the junctions in small vesicles. (M-R) Wing phenotypes implicating both cAMP effector pathways in Rab11 inhibition, from flies of the following genotypes: (M) wild-type (N) 1096GAL4>PKA*. (O) 1096GAL4>Rap1* (more similar to the EF phenotype, see panel Q). (P) 1096GAL4>Rap1*+PKA* (showing synergism between PKA* and Rap1*). (Q) 1096GAL4>EF. (R) 1096GAL4>EF+Epac^RNAi^. Knock-down of Epac expression significantly suppresses the EF phenotype. See S6 Fig showing quantifications of these phenotypes. In contrast, loss-of-function alleles of PKA-C1 do not reduce the EF phenotype significantly (S6 Fig).

cAMP stimulates two main effectors: PKA and Epac, a GEF that activates the small GTPase Rap1 [32] [33]. We activated each branch of the cAMP pathway separately, using either a constitutively active form of PKA (PKA*, consisting of the catalytic domain only [34]), or an activated form of Rap1 (Rap1*, which is locked in its GTP-bound form [35]). We previously reported that both PKA* and Rap1* expressed in the wing primordium caused a reduction in Rab11 levels, blocked apical accumulation of Delta, and prevented the formation of Sec15 structures in the wing primordium [23], suggesting that over-stimulation of each branch of the cAMP pathway can inhibit Rab11. We thought to resolve the respective activities of PKA and Rap1 on Rab11 function further, by co-expressing the activated form of Rab11 (Rab11*YFP) with either PKA* or Rap1*. PKA* profoundly altered Rab11* distribution, both by eliminating accumulation of Rab11* at AJs (Fig 2B and 2E, compare with 2A and 2D) in a similar, albeit stronger, fashion to EF (Fig 1F and 1I), and also by preventing the formation of secretory granules (or dramatically reducing their size). These combined effects of PKA* result in Rab11* being ubiquitously distributed throughout the cytoplasm (Fig 2B and 2E). A similar pattern was observed when staining for total endogenous Rab11, which lost its tendency to concentrate around the junctions in response to PKA* expression (Fig 2H, compare with 2G). Surprisingly, PKA* induced a strong increase in overall Rab11 levels in salivary glands, which is opposite to its effect in wing imaginal discs[19,23]. Consistent with Rab11-dependent trafficking being disrupted by PKA*, adherens junctions appeared weakened in PKA*-expressing glands, with more D-Ecad accumulating in the cytoplasm and around the AJs (Fig 2K) than in the wt glands (Fig 2J). In contrast to PKA*, Rap1* expression in salivary glands did not prevent Rab11*YFP from accumulating near cell boundaries (Fig 2C and 2F). However, instead of the typical single sharp line coinciding with cell junctions observed with Rab11*YFP alone (Fig 2D), co-expression with Rap1* resulted in a double row of Rab11* staining, revealing a narrow gap between adjacent cells (Fig 2F, arrows). This phenotype suggests a failure of the final fusion event between cargo vesicles and the plasma membrane. Consistent with these observations, endogenous total Rab11 staining was also concentrated in a sub-junctional zone in response to Rap1* expression (Fig 2I,arrows), revealing narrow intercellular gaps. These Rap1*-expressing glands also showed an accumulation of small D-Ecad-rich vesicles near inter-cellular boundaries, while normal AJs failed to form (Fig 2L). These results confirm the view that over-activation of each branch of the cAMP pathway can block Rab11-dependent trafficking, but that PKA* does so at an early step when vesicle loading takes place, while Rap1* acts later during the final vesicle delivery process.

In adult *Drosophila* wings, both PKA* and Rap1* cause phenotypes similar to that of EF (compare Fig 2N and 2O with 2Q) consisting of smaller wings with blisters and thicker veins. The PKA* phenotype, however, is predominantly restricted to the center of the wing (Fig 2N), while Rap1*, like EF, affects the entire wing blade (Fig 2O and 2Q). Consistent with PKA* and Rap1* intersecting a common pathway, we found that co-expression of Rap1* and PKA* led to a drastically enhanced synergistic phenotype (Fig 2P). While these gain-of-function studies reveal that both PKA and Rap1 signaling can interfere with Rab11 trafficking when artificially stimulated, we also tested which cAMP pathway might be required to mediate the effects of EF. We selectively blocked the Epac/Rap1 branch by expressing different EpacRNAi transgenes, which did not produce any notable phenotype on their own (S6 Fig). When combined with EF, however, EpacRNAi significantly reduced the EF phenotype (Fig 2R, compare with 2Q, see S6 Fig for quantifications). In contrast, reducing the levels of PKA-C1, the major PKA catalytic subunit in *Drosophila* (by two heterozygous loss-of-function PKA-C1 alleles), had little if any effect on the EF phenotype (S6 Fig). We conclude that the Epac/Rap1 pathway is the predominant mediator of EF in the wing epithelium.

### EF prevents association of Rab11* with its effectors

**Fig 3:**
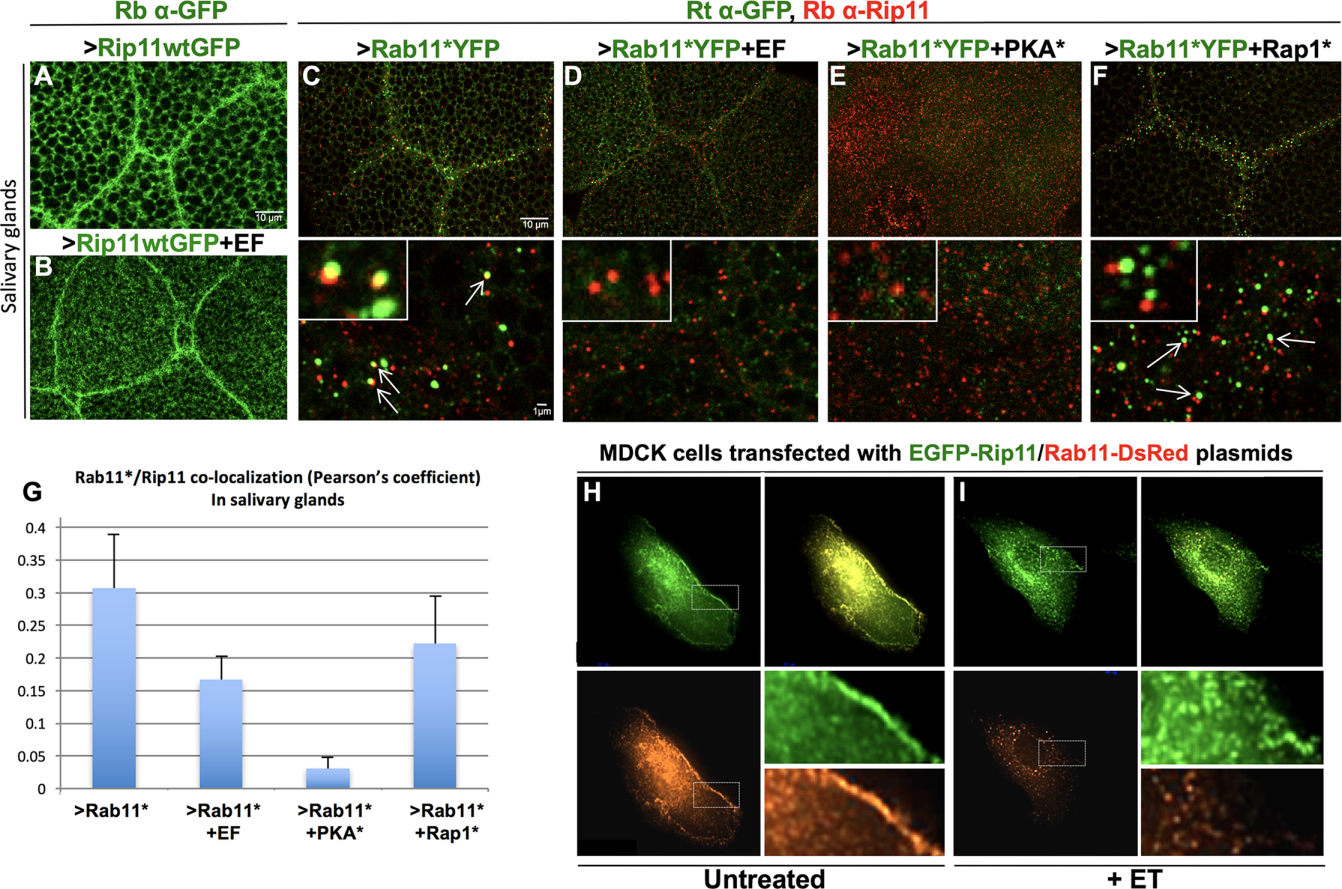
EF prevents association between Rab11* and its effector Rip11. (A) *Drosophila* salivary glands stained with a Rabbit anti-GFP antibody (Rb α-GFP), showing that dRip11GFP accumulates at cell-cell junctions in 1096GAL4>dRip11-GFP larvae. (B) 1096GAL4>dRip11-GFP+EF glands. Junctional staining is moderately weakened by EF. (C-F) Salivary glands co-stained with a Rat anti-GFP antibody (Rt α-GFP) and a Rabbit anti-dRip11 antibody (Rb α-Rip11), showing that the co-localization between Rab11* and dRip11 detected with this antibody combination is lost or reduced upon co-expression of Rab11*YFP with EF, PKA*, or Rap1*. Lower panels show higher magnifications. Insets show representative examples of vesicles with or without Rab11*/dRip11 co-localization. (C) 1096GAL4>Rab11*YFP, arrows indicate Rab11*/dRip11 co-localization. (D) 1096GAL4>Rab11*YFP+EF. (E) 1096GAL4>Rab11*YFP+PKA*. (F) 1096GAL4>Rab11*YFP+Rap1*. Arrows point to adjacent but non-overlapping punctae. (G) Quantification of Rab11*YFP/dRip11 co-localization in panels C-F measured by the Pearson’s coefficient. (H-I) MDCK cells transfected with constructs expressing human Rip11-GFP and Rab11wt-DsRed. (H) Untreated cells showing Rab11-Rip11 co-localization throughout the cell with higher levels of Rip11 and Rab11 at cell borders. (I) Rip11 and Rab11 no longer co-localize in cells treated with ET (Edema Toxin = EF+PA). While a minor component of Rip11 is still evident at cell borders, Rab11 fails to reach the cell borders.

In order to direct cargo vesicles to the AJs and promote their fusion with the plasma membrane, Rab11 must interact with several known effectors, including Rab11-FIPs (Rab11 Family-Interacting Proteins [36]) and Sec15, a component of the exocyst complex that is critical for its assembly [20]. *Drosophila* has a single ortholog of Rab11-FIP (dRip11 [37]), as well as unique representatives of all core exocyst components [18]. We first tested the effect of EF on Rab11 effectors by expressing a full length GFP-tagged dRip11 UAS transgene [37] in the salivary glands. When expressed alone, this fusion protein was strongly concentrated at cell junctions (Fig 3A). Co-expression of EF with dRip11 reduced, but did not eliminate junctional accumulation of dRip11 and also resulted in forked and irregular cell borders (Fig 3B). Because Rip11 is a Rab11-binding protein, we also examined association between these two proteins, which we visualized by expressing Rab11*YFP (detected with a rat anti-GFP antibody) and staining for the endogenous dRip11. This particular double stain revealed frequent co-localization of the two proteins in bright dots in the vicinity of intercellular junctions (Fig 3C, arrows in lower panel). Co-expression of EF with Rab11*YFP severely reduced its co-localization with Rip11 (Fig 3D and 3G), supporting the hypothesis that high levels of cAMP trigger a dissociation of Rab11 and Rip11, or prevent their initial association. Similarly, co-expression of PKA* with Rab11* also largely eliminated co-localization of Rab11* and Rip11 (Fig 3E and 3G). Interestingly, Rap1* also affected this association: Rab11*YFP and Rip11 proteins remained present in adjacent but non-overlapping vesicles (Fig 3F, quantifications in 3G), suggesting that both Rap1* and PKA* have effects on the Rab11*-dRip11 interaction albeit through distinct mechanisms.

In contrast to full-length dRip11, a truncated dominant-negative form of dRip11 (dRip11DN) retaining only the C-terminal Rab11-binding domain[37], did not accumulate at cell-junctions in salivary glands, consistent with its N-terminal cholesterol-binding domain being essential for associated cargo vesicles to traffic to AJs. Instead, dRip11DN was distributed in a reticulated pattern throughout the cytoplasm, although it did show higher juxta-junctional levels (S7 Fig). Small cytoplasmic Rab11 staining punctae strongly co-localized with dRip11DN-GFP (S7 Fig), consistent with dRip11DN retaining its Rab11-binding domain. Interestingly, when co-expressed with EF, this punctate co-localization was not reduced, but rather transformed into rings that encircled secretory granules (S7 Fig). Thus, EF does not abrogate association between Rab11 and dRip11DN. Because deletion of the first 700 aa of Rip11 (a region containing a verified PKA phosphorylation site in humans [38] and several such predicted sites in *Drosophila*) results in an EF-resistant association between Rab11* and dRip11DN, it is possible that PKA phosphorylation may contribute to this dissociation.

We next examined the relationship between Rip11 and Rab11 in mammalian Madin-Darby canine kidney (MDCK) cells, in which the role of Rab11 in cadherin trafficking has been well established [39]. Co-expression of human Rab11-DsRed and EGFP-Rip11 constructs in these cells revealed strong co-localization throughout the cytoplasm, and a tendency for both proteins to accumulate at cell margins (Fig 3H). Upon treatment with ET, however, we observed a significant reduction in Rab11 and Rip11 co-localization, and a reduction in Rab11 localization at the plasma membrane (Fig 3I, see S8 for Pearson’s coefficient quantifications). Mirroring our observations in *Drosophila* salivary glands, EGFP-Rip11 accumulation at cell boundaries was reduced by ET-treatment (Fig 3I). Interaction between endogenous activated Rab11 and its effectors was also tested in human brain microvascular cells (HBMECs) transfected with a mammalian Sec15-GFP construct. High-level expression of Sec15-GFP led to formation of punctate fluorescent structures (S9 Fig), the formation of which depends on Rab11 [19]. Consistent with Sec15 associating with the active form of Rab11, we detected, using an anti-Rab11* antibody, a high degree of co-localization between Sec15-GFP fluorescence and Rab11*. In this context of Sec15 over-expression, we also visualized co-localization of Rab11* with endogenous Rip11 (S9 Fig). When these cells were treated with ET, Sec15-GFP punctae were significantly reduced after 6 hours, and the remaining punctae no longer co-localized with Rab11* or Rip11 (S9 Fig). Cumulatively, these experiments suggest that EF-induced dissociation of Rab11* from its effectors Rip11 and Sec15 is a well-conserved process across species.

### Activation of the small GTPase Arf6 phenocopies aspects of EF treatment

**Fig 4:**
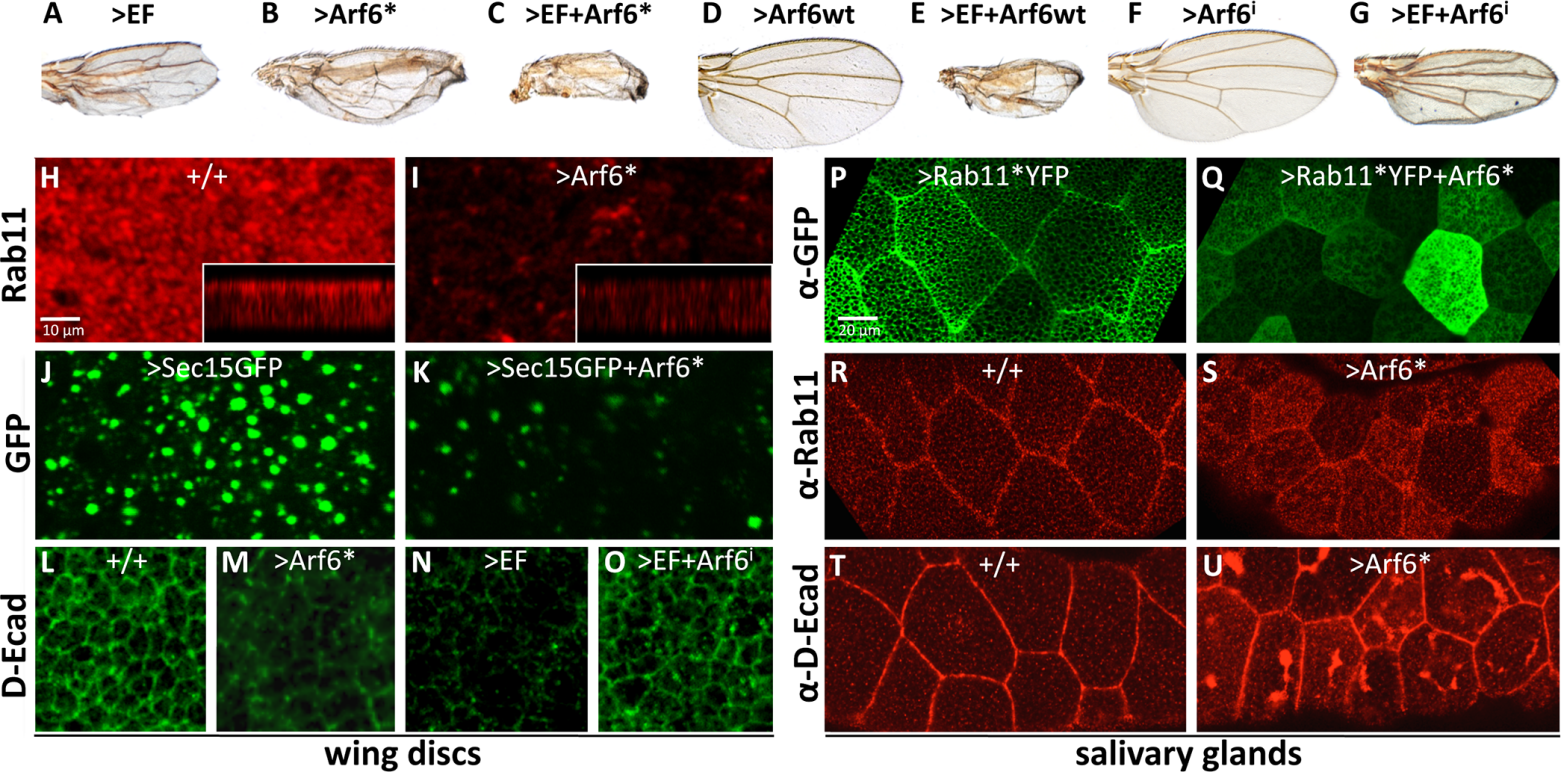
Activation of Arf6 partially mimics the effect of EF. (A-G) Wings showing EF and Arf6-dependent phenotypes. (A) 1096GAL4>EF. (B) 1096GAL4>Arf6*. Activated Arf6 (Arf6*) causes a phenotype very similar to that caused by EF. (C) 1096GAL4>EF+Arf6*, showing an additive phenotype. (D) 1096GAL4>Arf6wt wing, displaying a wild-type phenotype. (E) 1096GAL4>EF+Arf6wt wing revealing synergy between EF and Arf6wt. (H-I) Wing imaginal discs stained with an anti Rab11 antibody. (H) In wild-type discs, Rab11 displays a dotted apical distribution. (I) In discs expressing Arf6* (1096GAL4>Arf6*), Rab11 levels are reduced, and apical restriction is lost. (J-K) Wing imaginal discs expressing Sec15-GFP. (H) Sec15-GFP expressed at high levels in 1096GAL4>Sec15-GFP discs forms large fluorescent punctae. Like EF, Arf6* expression in (K) 1096GAL4>Sec15-GFP+Arf6* discs blocks formation of Sec15-GFP punctae and reduces Sec15-GFP levels. (L-O) D-Ecad staining in wing imaginal discs. (L) D-Ecad stain in wild-type discs, revealing the apical network of AJs. (M) In 1096GAL4>Arf6* discs, apical D-Ecad levels are severely reduced, an effect similar to that of EF (N). Arf6-RNAi suppresses the EF phenotype and partially restores normal D-Ecad at AJs (O). (P) A 1096GAL4>Rab11*YFP salivary gland showing Rab11* targeting to the AJs, revealed by an anti-GFP stain. (Q) Arf6* blocks Rab11* targeting to cell junctions in 1096GAL4>Rab11*+Arf6* glands. (R) Endogenous Rab11 stain of wild-type salivary glands. (S) Arf6* causes endogenous Rab11 to accumulate in the cytoplasm and diminishes its localization at junctions. (T) D-Ecad stain of wild-type salivary glands. (U) Arf6* results in accumulation of large intracellular inclusions of D-Ecad in 1096GAL4>Arf6* salivary glands.

Junctional homeostasis is also established by a balance of Rab11-mediated delivery of junctional cargo and retrieval of proteins via endocytic processes. Arf6, a small GTPase of the Arf subfamily (ADP-ribosylation factors) is involved in early steps of endocytosis from the plasma membrane, exocytosis, and endosomal recycling, and is predominantly localized to the plasma membrane and endosomes [25]. Arf6 activation contributes to sepsis by promoting vascular leakage through excessive internalization of VE-cadherins [40] and additionally interacts directly with exocyst components [41]. We tested whether Arf6 also exerted a role in mediating the phenotypes caused by cAMP-producing toxins in our system by expressing an activated form of this small GTPase (Arf6*). Strikingly, Arf6* caused a wing phenotype nearly identical to that induced by EF (Fig 4A and 4B) or Rab11DN [19], consisting of small narrowed wings with thickened veins and blisters. In contrast, the wild-type form of Arf6 (Arf6wt) when expressed alone did not cause any detectable phenotype (Fig 4D). However, both activated and wild-type forms of Arf6 strongly enhanced the EF wing phenotype (Fig 4C and 4E). Further analysis revealed that Arf6* reduced the levels and apical restriction of Rab11 in the wing discs (Fig 4H and 4I), diminished the formation of Sec15-rich structures, and reduced total Sec15 levels (Fig 4J and 4K), in a manner similar to what we observed with EF [19]. Arf6* expression also reduced the levels of junctional and total D-Ecad (Fig 4M, compare to 4L), as would be expected from its wing phenotype and effects on Rab11 and Sec15. Given the striking similarities between Arf6* and EF phenotypes, we tested whether Arf6 contributes to mediating the effect of EF in the developing wing, making use of an Arf6-RNAi construct that is highly effective in suppressing Arf6 expression (S2 Fig). Arf6-RNAi did not produce any noticeable phenotype on its own (Fig 4F), but did exert a significant suppression of the EF wing phenotype (Fig 4G, compare with 4A). Arf6-RNAi suppression of the EF phenotype was yet more pronounced at the level of junctional E-Cadherin expression (Fig 4O, compare to 4N). We also tested whether Arf6* altered the distribution of Rab11*YFP in salivary glands. As observed with EF (Fig 1F and 1I), Arf6* reduced the concentration of Rab11*YFP at the AJs (Fig 4Q, compare to 4P), revealing that Arf6* similarly inhibits Rab11 at a step subsequent to GTP loading. In contrast to EF, however, Arf6* induced an intracellular accumulation of Rab11, while also reducing Rab11 levels near the junctions (Fig 4S, compare to 4R), and caused striking accumulations of D-Ecad below the apical plasma membrane (Fig 4U, compare to 4T). In aggregate, these observations suggest that the Arf6 pathway inhibits Rab11 activity, but does so through a mechanism distinct from that of EF.

### Inhibitors of either Arf6 or Epac protect against ET in human cells and *in vivo*

**Fig 5:**
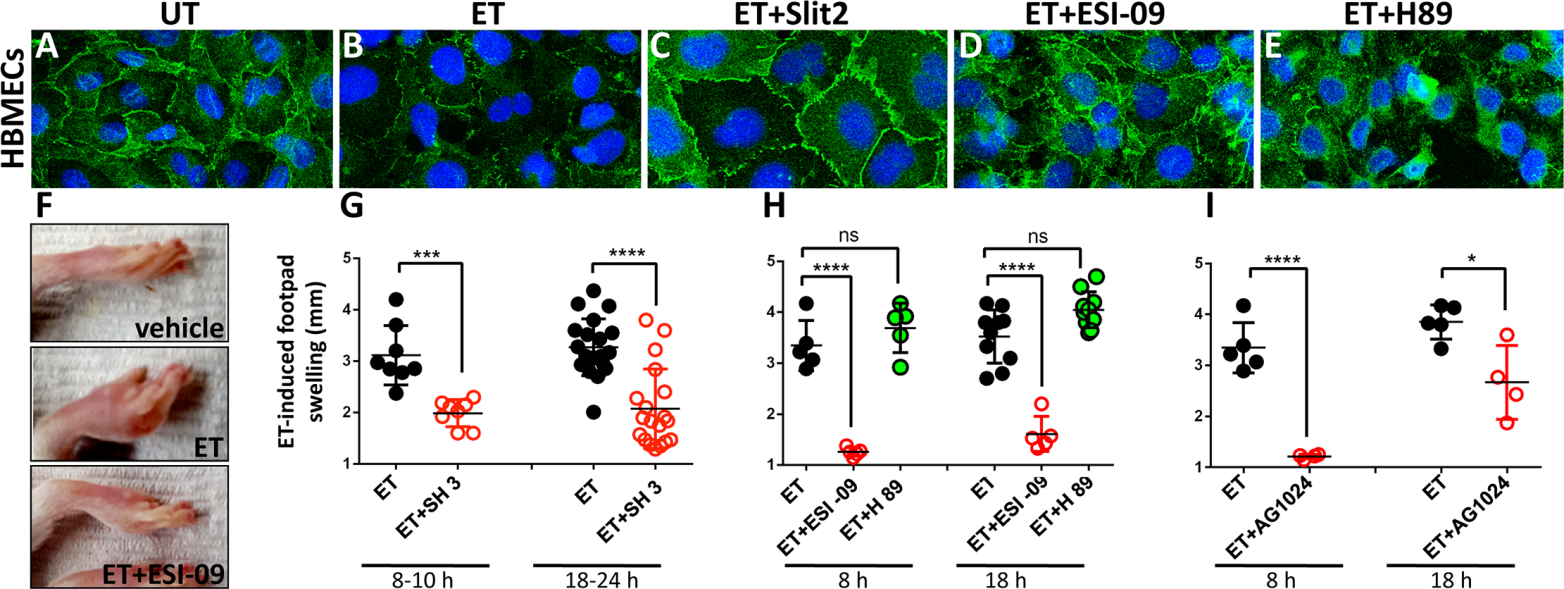
Arf6 and Epac/Rap1 play key roles in mediating EF toxicity in mammalian systems. (A-E) HBMECs (Human Brain Microvascular Endothelial Cells) stained with an anti pan-Cadherin antibody. (A) The pan-Cadherin (p-Cad) stain clearly delineates cell borders in untreated cells (UT). (B) ET (EF+PA) treatment results in strong reduction in Cadherin staining and loss of AJs. (C) Co-treatment of cells with ET and the Slit2 peptide, which prevents Arf6 activation via induction of ArfGAP activity, fully restores AJs compromised by ET treatment. (D) Co-treatment with the Epac-specific inhibitor ESI-09 partially rescues p-Cad expression at AJs. (E) Co-treatment with the PKA-inhibitor H89 does not appreciably rescue junctional expression of pCad at cell borders but does restore intracellular p-Cad staining (F-I) ET-induced footpad swelling (edema) can be suppressed by pharmacological intervention. (F) ET-injected footpads develop a striking edema. Pre-treatment with ESI-09 consistently blocks this symptom. Pre-treatment with (G) SecinH3, a compound that indirectly suppresses the activation of Arf6 (****p<0.0001, ***p<0.001), and (H) the Epac pathway inhibitor ESI-09, but not the PKA inhibitor H89, abrogate EF-induced edema (****p<0.0001, ns non significant). (I) AG1024, a compound that may indirectly block Rap1 activation, acts as a potent inhibitor of ET-induced edema at early times, but offered more modest protection at a later time point (****p<0.0001, *p<0.05).

As described above, activation of PKA*, Rap1*, and Arf6* mimic features of the EF phenotype in *Drosophila*. We wondered whether the same might be true in mammalian systems and thus examined the relative contributions of each of these pathways in EF-induced toxemia in various experimental models relevant to *B*.*a*. infection in mammals. In HBMECs, ET treatment reduced total cadherin levels and weakened AJs as indicated by staining with an anti-pan-cadherin (p-Cad) antibody (Fig 5B, compare with 5A), as shown previously [19]. Western-blot analysis confirmed a drastic decrease in p-Cad and Rab11 levels in response to treatment with ET or dcAMP (S11 Fig). Similar reductions in Rab11 levels in response to EF have been documented histochemically in *Drosophila* wing imaginal discs [19]. To inhibit the Arf6 pathway, we treated HBMECs with Slit2, a secreted peptide that activates the Robo4 receptor to promote vascular stability via stimulation of the ArfGAP GIT[28]. Cells co-treated with ET and Slit2 appeared resistant to ET, as clearly illustrated by the robust rescue of junctional pan-cadherin accumulation (Fig 5C). These findings suggest that Arf6 contributes to EF-induced inhibition of the Rab11/exocyst complex and weakening of AJs. Next, to determine the relative contribution of each branch of the cAMP pathway, we co-treated ET-intoxicated cells with ESI-09, an inhibitor specific for Epac[26], or with H89, a well-characterized inhibitor of PKA[42]. We found that only ESI-09 could partially restore cadherin expression at AJs (Fig 5D), although junctions did not appear as regular as in untreated cells. In contrast, H89 provided no obvious rescue to the ET-induced junctional phenotype (Fig 5E). We conclude that Epac/Rap1 is the predominant pathway mediating the effects of ET on exocyst-dependent junctional cadherin trafficking in HBMECs.

**Fig 6:**
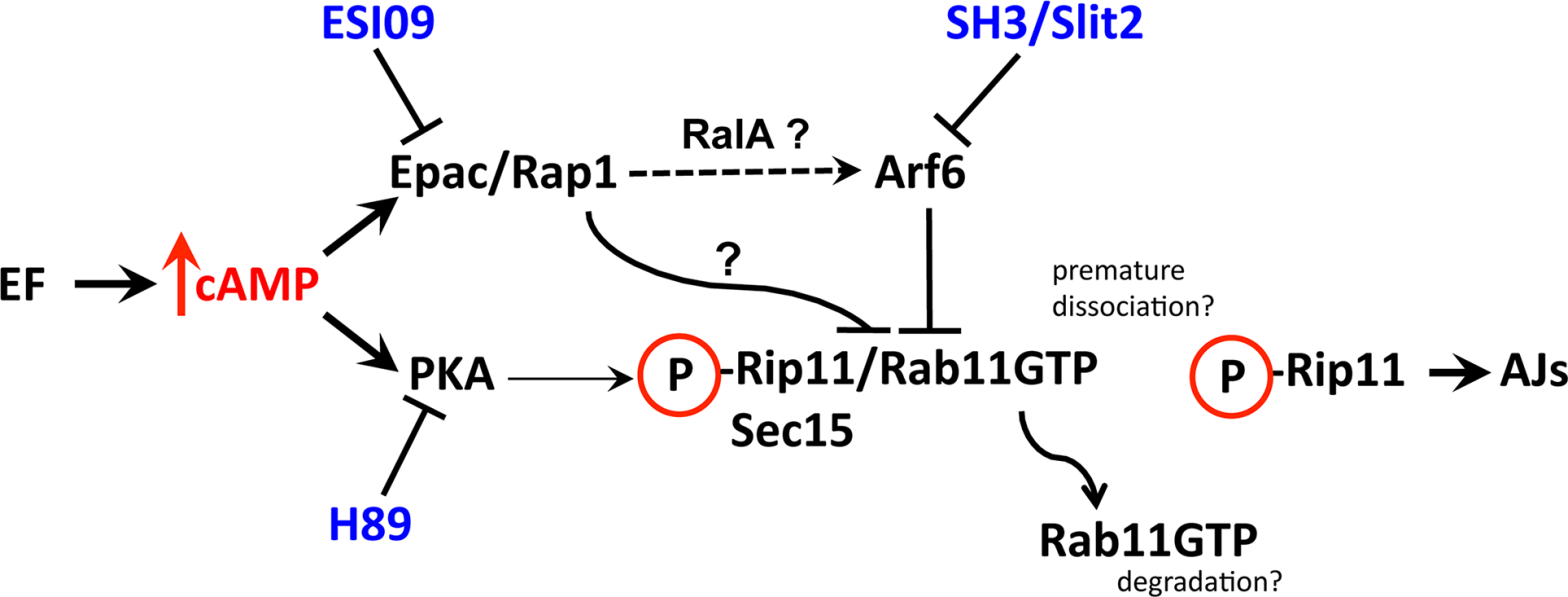
Summary diagram. EF-induced cAMP overload can activate either or both of the PKA and Epac/Rap1 effector pathways depending on biological context. In this model, uncontrolled cAMP production by EF leads to activation of PKA and/or Epac. PKA stimulation promotes dissociation of Rab11 from its effectors Rip11 and Sec15. This premature dissociation may prevent the activated form of Rab11 from reaching the AJs, and thereby block delivery of cargo proteins (e.g., Cadherins and Notch ligands). In some cellular contexts, dissociation of Rab11-GTP from Rip11 may also lead to Rab11 degradation (e.g., *Drosophila* wing discs and human endothelial cells), while in others, only to the loss of junctional accumulation (e.g., *Drosophila* salivary glands). Over-activation of Rap1 by Epac leads to inhibition of exocyst-mediated vesicle fusion at the cell surface. Epac/Rap1 may act indirectly via sequential activation of RalA and Arf6, which cross inhibits Rab11-mediated cargo delivery to junctions, or directly through an unknown mechanism. The relative contribution of each cAMP-responsive pathway may depend on cell type and organism, although, the Epac/Rap1 branch (blocked by ESI09, and possibly by AG1024) appears to be the primary mediator of EF in fly wings, human endothelial cells, and mouse footpads. The Arf6 GTPase inhibits Rab11* targeting to the AJs, an effect that may be mediated by its interaction with exocyst components. Inhibition of Arf6 by Slit2 or Secin H3 provides protection against EF in cultured humans cells and *in vivo* in mice, respectively.

We next examined the relative contributions of the PKA and EPAC pathways as well as Arf6 in a quantitative *in vivo* footpad-swelling assay, in which intra-dermal injection of ET results in a robust and quantifiable edema (Fig 5F) [43]. In mice pretreated with SecinH3, a compound that inhibits the Arf6-GEF ARNO thereby lowering Arf6 activity [28], ET-induced swelling was strongly reduced (Fig 5G). Indeed, in animals in which systemic pre-treatment with the drug induced observable symptoms of malaise (presumably indicative of potent systemic pharmacological action), ET-induced edema was virtually abolished. We then examined contributions of the cAMP effector PKA and Epac to ET-induced edema, by comparing the relative abilities of H89 and ESI-09 to block ET-induced footpad swelling (Fig 5F and 5H). Reinforcing the results of our experiments in flies and with HBMECs, we found that while ESI-09 virtually abolished ET-induced edema, H89 had little or no effect (Fig 5H). We conclude that the Epac/Rap1 pathway is the primary mediator of EF-induced edema. In addition, we tested the effect of AG1024 [44], an inhibitor of insulin-like growth factor receptor (IGF-1R) [45]. Because IGF-1R has been shown to indirectly stimulate Rap1 [46], we hypothesized that inhibition of IGF-1R by AG1024 might conversely result in Rap1 inhibition. Indeed, pre-treatment of mice with AG1024 also led to significant reduction of edema, which was particularly strong at early time points (Fig 5I). These findings, together with results described above provide a framework for how effector pathways contribute to cAMP-mediated disruption of Rab11-dependent membrane trafficking (See Fig 6 for summary diagram). In addition to a prominent role of the Epac/Rap1 branch in mediating the effect of ET, our study reveals a previously unappreciated form of negative cross-regulation between the machineries responsible for the delivery versus retrieval of membrane bound cargo. Importantly, small molecule inhibitors such as SecinH3, ESI-09, and AG1024 offer potential for new therapeutic avenues for treating a range of diseases involving compromised barrier integrity of epithelial or endothelial sheets.

## Discussion

In previous studies, we established that two cAMP toxins, EF from *Bacillus anthracis* [19] and Ctx from *Vibrio cholerae* [23], block Rab11-mediated endocytic recycling of cargo such as signaling ligands and adhesion proteins (reviewed in [15,16]), ultimately leading to inhibition of Notch signaling and loss of barrier integrity. However, the precise mechanisms by which cAMP overproduction interfered with Rab11-dependent trafficking remained to be explored. Here, we examined how cAMP effector pathways converge on discrete nodes of the trafficking process subsequent to the GTP loading step to efficiently interrupt endocytic recycling.

### EF disrupt Rab11-dependent trafficking after the GTP loading step

As is typical of small GTPases, Rab11 cycles between active (GTP-bound) and inactive (GDP-bound) conformations, the former permitting interaction with effector proteins to carry out downstream functions. Two types of regulators, activating GEFs and inactivating GAPs provide control for this essential cycle. In the particular case of Rab11, Crag (the *Drosophila* homolog of human DENND4A) is the only known Rab11-dedicated GEF [31]. Similarly, only one Rab11-specific GAP has been identified: EVI5 [47–49]. Neither of these regulators contains an identified cAMP-binding domain that could provide a direct link between cAMP and upstream regulation of Rab11. Consistent with this inference, we found that EF acted on Rab11 at a step subsequent to GTP loading. Indeed, transport of vesicles carrying the constitutively activated mutant Rab11*YFP were blocked by EF, while total endogenous levels of Rab11-GTP did not appear to be greatly altered.

### EF blocks interaction between Rab11 and its effectors Rip11 and Sec15

Association between Rab11 and its effectors Rip11 and Sec15 was abrogated by EF in several settings, including *Drosophila* salivary glands and human cells. The Rab11 effector Rip11 is an attractive candidate for mediating some of EF effects, as it contains a verified PKA phosphorylation site located in the central portion of the protein [38]. Indeed, PKA-dependent phosphorylation of Rip11 is required for cAMP-potentiated insulin secretion in pancreatic β-cells [38]. In addition, Ser/Thr phosphorylation is responsible for Rip11 transition from the insoluble to cytosolic fraction in intestinal CACO-2 cells [50]. Although it was not determined whether the latter modification was specifically PKA-dependent, this study proposed a model in which phosphorylation of Rip11 is essential for cycling to a free state following interaction with Rab11 and specific membrane compartments prior to its re-associating with Rab11. Our data show that the association between Rab11 and Rip11 can be disrupted by EF in *Drosophila* and mammalian endothelial or embryonic kidney cells. It is possible that unrelenting phosphorylation of Rip11 by PKA may cause the premature dissociation of Rab11 and its effectors, potentially leading to a failure to reach the AJs. While this PKA-dependent phosphorylation of Rip11 has been demonstrated in human pancreatic cells, it is not known whether it occurs in *Drosophila*. As dRip11 contains 19 candidate PKA phosphorylation sites, further investigation will be necessary to determine whether phosphorylation of one or more of these sites occurs and promotes the dissociation between dRip11 and Rab11. Intriguingly, *Drosophila* Sec15 also harbors several putative PKA phosphorylation sites, although such predicted sites are missing in its human counterpart. Importantly, we found that artificial stimulation of Rap1 also causes a loss in Rab11*/Rip11 co-localization resulting in correlated but separated staining foci of these two proteins, suggesting that the later acting Epac/Rap1 pathway may feedback on this process (see below).

### Essential contribution of the Epac/Rap1 branch of the cAMP pathway

The second branch of the cAMP pathway mediated by the cAMP-regulated GEF Epac and its partner Rap1 [32] contributes significantly to the effect of EF in flies, and surprisingly appears to play the predominant role in the mammalian systems we examined. In flies, activated Rap1 (Rap1*) causes a wing phenotype more similar to that of EF and Rab11DN than that of PKA*. We previously reported that Rap1* reduces the levels of Rab11 and prevents formation of Sec15 punctae [23]. In the present study, we find that blocking expression of Epac significantly reduces the intensity of the EF phenotype. In addition, Rap1* alters the distribution of Rab11* and inhibits Rab11*/Rip11 co-localization. We hypothesize that the final exocyst- and SNARE-dependent fusion event with the apical plasma membrane is subjected to inhibition by exuberant Rap1* activity, leading to accumulation of non-functional Rab11* just beneath the plasma membrane. Consistent with this hypothesis, Rap1 has been implicated by many studies in regulating of both cadherin and integrin-mediated cell-cell adhesion (reviewed in [51] [52] [53]). Further indicating a functional connection between Rap1 signaling and Rab11-dependent trafficking, Rap1 and Rab11 over-expressed in human cells co-localize in a recent study [54]. Additional experiments will be required to elucidate the molecular interactions connecting the activities of these two GTPases. The small GTPase RalA is a possible candidate for mediating the activity of Rap1, through activation of the Rap1 effector Rgl1, a positive regulator (GEF) of RalA. Because lowering the dose of Rgl1, or expressing a dominant-negative form of RalA, can suppress Rap1*-induced phenotypes in *Drosophila*, it has been proposed that RalA may act downstream of Rap1 [35]. Also, RalA is known to directly bind to exocyst components Sec5 [55] [56] [57] and Exo84 [58] and plays a central role in regulating exocyst-mediated processes in several settings, including the release of Von-Willebrand Factor from endothelial cells, or insulin secretion in pancreatic β-cells (reviewed in [51] and [59]). In addition, a recent study identified Arf6 as a key component acting downstream of RalA, mediating its effect on exocyst-dependent delivery of raft micro-domains to the plasma membrane [60]. Thus, RalA over-activationmay contribute to mediating the effect of cAMP toxins on exocyst inhibition downstream of Rap1, although this hypothesis needs to be tested in future experiments.

### EF causes a loss in Rab11 and cadherin levels

We previously showed that EF caused a drastic reduction in total Rab11 levels in wing epithelial cells[19]. Here, we find that this effect is also evident in HBMECs treated with ET, but is dependent on cell context, since inhibition of Rab11 function can be uncoupled from reduction in total Rab11 levels in *Drosophila* salivary glands. This reduction in Rab11 levels is unlikely to derive from transcriptional inhibition, as infection of HBMECs with *B*. *a* Sterne did not result in any change in levels of Rab11 transcripts (Nina Van Sorge, personal communication). Similarly, in *Drosophila* wings, where EF also triggers great reduction in Rab11 protein levels, mRNA transcript levels again were not greatly affected (Valentino Gantz, personal communication). In HBMECs, where Rab11 levels are reduced by ET treatment, we observed that total levels of cadherins were also severely reduced in ET-treated cells. Although the precise mechanism responsible for the loss of these proteins following ET treatment remains to be explored, it is worth noting that degradation of VE-cadherins has been observed following silencing of Rab11 in human endothelial cells [61], in which Rab11 is important for stabilizing cadherins at the AJs. Thus, it is possible that following EF intoxication, Rab11 and cadherins are routed to the lysosomal pathway and degraded, further impairing endocytic recycling and junctional integrity. Such an attractive hypothesis could explain the catastrophic loss of cadherins observed in ET-treated cells.

### The “cAMP paradox”

Numerous studies have demonstrated the positive role of physiological induction of cAMP in junction establishment and stabilization, through stimulation of both PKA and Epac [38,62]. It may therefore seem counterintuitive that cAMP produced by EF or other toxins may exert an opposing effect and jeopardize junctional integrity. In principle, high versus low concentrations, sustained versus transient production, and perinuclear vs cortical subcellular distribution of toxin-delivered cAMP could elicit such opposite outcomes. In the particular case of Rab11-dependent trafficking, low physiological levels of cAMP may exert their positive effects by promoting the release of Rip11 from Rab11, as necessary to allow the final fusion event between recycling endosomes and the plasma membrane. In contrast, pathologically elevated cAMP concentrations may cause premature dissociation of the Rab11-Rip11 complex and permanently block that cycle. Similarly, uncontrolled stimulation of Rap1 by Epac could also have a negative impact on junctional transport: titration of critical partners, failure to return to complete the necessary GTP/GDP cycle, or negative feedback interference with other important steps, could explain the occurrence of this apparent paradox. Another molecule potentially at play during the response to cAMP is the small GTPase RhoA. RhoA can be phosphorylated by PKA, which inhibits its activation and prevents increased endothelial permeability during inflammation [63], the potential interplay between RhoA and the exocyst downstream of cAMP signaling in EF-intoxicated cells also merits further examination.

### Integration of membrane trafficking delivery and retrieval pathways

The small GTPase Arf6 initiates retrieval of membrane proteins from cell junctions in a wide variety of cells types [25]. Arf6, a member of the ADP-ribosylation factor subfamily, is located at the plasma membrane and some endosomal compartments, and is involved in endocytosis from the plasma membrane, vesicular recycling, and exocytosis [64]. Importantly, Arf6 plays a role during sepsis to mediate acute VEGF-induced vascular permeability [40,65]. Whether linchpin regulators of opposing vesicular trafficking pathways such as Arf6 and Rab11 interact had not yet been extensively explored. In this study, we present evidence that these trafficking systems do in fact engage in cross-inhibitory interactions. Consistent with the published role of Arf6 in promoting VE-cadherin endocytosis [66], the activated form of Arf6 (Arf6*) caused phenotypes similar to those of EF. Our findings suggest that the activity of Arf6 negatively feeds back on vesicular transport to the plasma membrane by inhibiting Rab11 function. Previous studies showed that Arf6 physically interacts with the exocyst component Sec10 [41], defining a possible avenue for our observed effects of Arf6 on Rab11 levels and distribution. Given the negative regulation of Rab11 by Arf6 in flies and its known role in compromising barrier function in the mammalian vasculature during sepsis [28,40], we tested whether inhibitors of this pathway might antagonize the effects of EF. In human endothelial cells, we indeed found that treatment with Slit2, a secreted peptide indirectly blocking Arf6 function, could reverse the effects of EF, restoring junctional integrity. Similarly, pharmacological inhibition of Arf6 by SecinH3, a compound that inhibits the ArfGEF ARNO, potently blocked EF-induced edema in a mouse footpad assay.

An emerging lesson from the current and prior studies is that blocking multiple steps of branching pathways that converge on critical nodes in endocytic recycling may allow pathogens to weaken host protective mechanisms that rely on junctional integrity [24]. For example, LF, the other toxic factor secreted by *B*.*a*, blocked exocyst-mediated vesicular docking downstream of Rab11 via inhibition of MAPK signaling. It will be interesting to explore how the various effects of EF and LF are integrated to achieve an efficient inhibition of junctional delivery, and if any compound identified in this study can also block some of the downstream effects of LF. Altogether, our study suggests that a broad range of barrier disruptive diseases ranging from cAMP related toxemia to inflammatory autoimmune diseases that involve positive feedback loops between immune activation and barrier disruption, could potentially be treated with compounds that inhibit Arf6 or Epac/Rap1, or by yet undiscovered compounds that may boost Rab11 activity.

## Materials and methods

### Ethics statement

All experiments were performed in strict accordance with guidelines from the National Institute of Health and the Animal Welfare Act, approved by the Animal Care and Use Committee of University of California, San Diego and the National Institute of Allergy and Infectious Diseases, National Institutes of Health (approved protocols s00227m and LPD-8E). Anesthesia and euthanasia were performed using Isoflurane and CO2, respectively. All efforts were made to minimize suffering of animals employed in this study.

### *Drosophila* genetics

UAS-EF construct and line were described previously[19,23]. UAS-Rab11wtYFP/TM3 (#9790), UAS-Rab11*YFP (3^rd^ chr. # 9791), UAS-Rab11DNYFP (#23261), UAS-CragRNAi (2^nd^ chr. #53261), UAS-CragHA (3^rd^ chr. #58463), UAS-Arf6RNAi (3^rd^ chr. #51417), UAS-Pka^DN^/CyO (#5282), and Pka-C1^B10^ (#32018) lines were obtained from Bloomington Drosophila Stock Center (BDSC). UAS-EpacRNAiv50272 and UAS-EpacRNAiv50273 were obtained from Vienna Drosophila Resource Center (VDRC). UAS-Rap1*/TM6 and UAS-PKA*/CyO were generated by I. Hariharan (UCB), and D. Kalderon (Columbia University), respectively. UAS-Rip11GFP and UAS-Rip11DNGFP were kindly provided by Don Ready (Purdue University). UAS-Arf6* was generated in the Olson laboratory (UT Southwestern).

### Immunological stains of wing discs and salivary glands

Imaginal discs were dissected, fixed and stained using standard procedures. Salivary glands were dissected similarly, fixed for 30 minutes, and left attached to carcasses until ready to mount in SlowFade (LifeTechnologies #S36936), using double sided tape as a spacer to prevent tissue squashing. Antibodies: rabbit anti-GFP antibody (1/500, ThermoFisher #A6455), rat-anti GFP antibody (1/500, SCBT #sc-101536), mouse anti-Rab11 (1/200, BD Biosciences #610657), mouse anti Rab11-GTP (1/100, NewEast Biosciences #26919), D-Ecad (1/500, DSHB #DCAD2). The rabbit anti-Rip11 (1/1000) was a gift from D. Ready (Purdue University) and A. Satoh (Hiroshima University, Japan), and guinea pig anti-Sec15 (1/1000) was kindly provided by Hugo Bellen (Baylor College of Medicine). Images were collected by confocal microscopy on a Leica TCS SP5. All images were acquired using a 40X or 63X objective, and all higher magnifications were obtained using a 4X digital zoom. Co-localization quantifications in Fig 3 used the coloc2 tool in ImageJ.

### Transient expression of DsRed-Rab11A and Rip11-EGFP in MDCK cells

MDCK cells (ATCC CCL-34) were maintained in DMEM (Corning; Manassas, VA) containing 10% FBS, 1% Penicillin/streptomycin, 2 mM L-glutamine and were incubated in 37°C, 5% CO_2_ atmosphere. Cells were gently dislodged with 0.05% trypsin (Mediatech) and were electroporated with cDNA expressing DsRed-Rab11A (Addgene) and Rip11-EGFP (Kind gift from Dr. Rytis Prekeris, Univ. of Colorado, Denver) using Neon Transfection system (Life Technologies) according to manufacturer’s protocol. Briefly, cells were rinsed once with PBS and resuspended at a density of 10^7^ cells/ml. cDNA expressing DsRed-Rab11A and Rip11-EGFP were added to the suspension, and cells were electroporated with a 10 μl Neon tip at 1650 V, 20 ms width and 1 pulse. Cells were transferred to 600 μl pre-warmed medium of which 300 μl cell suspension was plated on each well of 8 chamber tissue culture treated glass slide (BD Falcon, Bedford, MA). Cells were treated with 10 μg/ml EF +20 μg/ml PA for 4 h before fixation with 4% para-formaldehyde in PBS for 30 min at 37°C and processed for imaging. Fluorescence images were collected using a Delta Vision RT microscope. Colocalization between Rab11 and Rip11 was determined by measuring the Pearson's correlation coefficient (PCC) using the Velocity 6.3 imaging and analysis software (PerkinElmer). Costes automatic thresholding method [67] was applied for background discrimination.

### HBMEC intoxications and Sec15GFP transfections

HBMEC cultures were maintained in DMEM (Corning) containing 10% FBS, 1% Penicillin/streptomycin, 2 mM L-glutamine, and were incubated in 37°C, 5% CO_2_ atmosphere. Cells were gently dislodged with 0.05% trypsin (Mediatech Inc.) and cultured on glass poly-D-lysine coated chamber slides (BD Falcon #354108). At about 80% confluence, EF and PA (0.2 μg/ml and 0.4μg/ml, respectively) were added to cells. Drug co-treatments included: Slit2 (10μg/ml, R&D systems # 8616), ESI-09 (TOCRIS #4773, 100μM), and H89 (TOCRIS #2910, 10μM). After 24 h (Fig 5), cells were fixed for 10 min at -20°C in 100% Methanol, then washed with 0.1% Triton in PBS. Cells were stained with a mouse anti pan-Cadherin antibody (Abcam, clone CH-19, 1/100). For S6 Fig, transfection of the Sec15-GFP was performed with the FuGENE 9 transfection reagent (Roche) according to manufacturer recommendations. Cells were treated with ET (2 μg/ml EF and 4 μg/ml PA), and fixed after 6hrs of treatment for 30 mins in 4% paraformaldehyde in PBS. Cells were stained with rabbit anti Rip11 (Novusbio #NBP1-81855, 1/500) and mouse anti Rab11-GTP (NewEast Bioscience #26919, 1/100) antibodies overnight at 4°C. Coverslips were washed, and incubated with secondary antibodies before mounting with Prolong Gold with DAPI mounting media (ThermoFisher).

### Mouse footpad edema studies

BALB/cJ mice (8–10 weeks old, female; Jackson Laboratories) were intraperitoneally injected with drugs. SecinH3 (TOCRIS #2849), AG1024 (Selleckchem, S1234), ESI-09 (TOCRIS #4773) or H89 (TOCRIS #2910), or with vehicle (70% DMSO in isotonic glucose for SecinH3, 30% DMSO in isotonic glucose for other drugs) 2–3 h prior to injection of ET (0.15 μg/20 μl, right footpad) or PBS (20 μl, left footpad), and in the case of SecinH3, also 2 h post toxin injection. ESI-09, AG1024 and H89 were administered at 10 mg/kg and SecinH3 was administered as 250 μl of 2.5 mM solution. Edema was assessed at 8–10 h, and 18–24 h by dorsal/plantar measurements using digital calipers.

### Immunoblot analysis

Untreated or ET intoxicated (24h) HBMEC cells were lysed in RIPA buffer (Cell Signaling Technology) supplemented with mammalian protease inhibitor cocktail (Sigma Aldrich). The lysates were clarified by centrifugation at 1000 g for 10 min at 4°C and LDS sample buffer (NuPAGE) was added. Samples were boiled at 95°C, run on a 4–12% SDS polyacrylamide gel (Life Technologies) and transferred onto PVDF membrane (Bio-RAD). After incubation with primary antibodies against Rab11 (#71–5300, Thermo Scientific), Cadherins (CH-19, Abcam #ab6528), and actin (sc69879, Santa Cruz Biotechnology), blots were probed with respective HRP conjugated secondary antibodies and developed using SuperSignal West Pico chemiluminescent substrate (Thermo Scientific).

## Supporting information

S1 Fig Activated Rab11 (Rab11*) preferentially accumulates at AJs in *Drosophila* wing imaginal discs. (A-C) Expression of Rab11wt in 1096GAL4>Rab11wtYFP wing discs. (A) Rab11wtYFP detected with a rabbit anti-GFP antibody appears as a peppered stain near the apical surface. (B) D-Ecad/GFP double stain. (C) corresponding D-Ecad stain marking AJs. (D-F) Expression of Rab11* in 1096GAL4>Rab11*YFP wing discs. (D) Rab11*YFP detected with a rabbit anti-GFP antibody. In addition to a peppered apical stain, Rab11* shows a distinctive net-like pattern at cell borders. (E) D-Ecad/GFP double stain, revealing that Rab11*YFP tends to accumulate at the AJs. (F) Corresponding D-Ecad single stain.
(TIF)

S2 Fig RNAi transgenes specifically block expression of cognate proteins Sec15, Crag, and Arf6. (A) A 1096GAL4>UAS-Sec15GFP salivary gland, stained with an anti-GFP antibody. (B) Sec15GFP expression in strongly inhibited by co-expression of a Sec15^RNAi^ construct. (C) A 1096GAL4>UAS-CragHA salivary gland stained with an anti-HA antibody. (D) CragHA expression is suppressed by co-expression of a Crag^RNAi^ construct. (E) A 1096GAL4>UAS-Arf6Myc gland stained with an anti-Myc antibody. (F) Arf6Myc expression is blocked by co-expression of an Arf6^RNAi^ construct.
(TIF)

S3 Fig Blocking expression of Sec15, but not of the Rab11GEF Crag, prevents Rab11*YFP targeting to cell junctions in *Drosophila* salivary glands. (A-C) Rab11*YFP detected with a rabbit anti-GFP antibody in salivary glands. (A) Rab11*YFP selectively accumulates at the AJs in 1096GAL4>Rab11*YFP salivary glands. (B) Rab11* distribution is unchanged in 1096GAL4>Rab11*YFP+Crag^RNAi^ glands. (C) Rab11*YFP fails to accumulate at the AJs in 1096GAL4>Rab11*YFP +Sec15^RNAi^ salivary glands.
(TIF)

S4 Fig EF prevents Rab11* accumulation at AJs. Images from experiment described in Fig 1, panels E, F, H and I, were analyzed to quantify the effect of EF on junctional accumulation of Rab11*. Individual image crops from intercellular boundaries were generated. For each crop, average fluorescence was determined in ImageJ, and normalized to the average fluorescence found inside the corresponding cell. EF expression significantly reduces Rab11* accumulation at intercellular borders, (p<0.0001).
(TIF)

S5 Fig Inhibition of Rab11 function in salivary glands leads to abnormal accumulation of D-Ecad around AJs, and intercellular gaps. (A-D) Salivary glands stained with an anti-D-Ecad antibody. (A) A wild-type salivary gland showing D-Ecad accumulation at AJs. (B) A SglGAL4>Rab11DN salivary gland, in which Rab11 inhibition in this tissue leads to D-Ecad accumulation in broad zones around intercellular gaps. (C-D) Higher magnifications. (C) A wild-type salivary gland showing D-Ecad forming AJs (arrows). (D) A SglGAL4>Rab11DN salivary gland, revealing gaps between cells, and broad accumulation of D-Ecad around them (arrows). D-Ecad fails to form AJs.
(TIF)

S6 Fig Reduction of Epac -but not PKA- levels, suppresses the EF wing phenotype. (A-F) wings of the following genotypes: (A) Wild-type (+/+). (B) 1096GAL4>EF. (C) 1096GAL4>Epac^RNAi^. (D) 1096GAL4>EF+Epac^RNAi^. Inhibition of Epac expression potentlyreduces the EF phenotype. (E) PKA-C1^B10/+^ (B10 is a loss -of-function allele of PKA). (F) 1096GAL4>EF; PKA-C1^B10/+^. Reduction of PKA-C1 levels, either in a heterozygote loss-of-function PKA-C1 alleles (B10/+) or in flies expressing a dominant negative form of PKA-C (C1-DN), does not obviously alter the EF phenotype. (G) The surface areas of wings of the indicated genotypes were measured in Photoshop. Results were plotted as a histogram, with relevant p-values indicated. EF expression reduces wing size significantly compared to widl-type (wt) (****p<0.0001). Epac^RNAi^ ameliorates the EF phenotype (****p<0.0001).
(TIF)

S7 Fig EF does not disrupt dRip11DN/Rab11 co-localization in salivary glands. (A-C) 1096GAL4>Rip11DN-GFP salivary glands, stained with (A) a rabbit anti GFP antibody, (B) a mouse anti Rab11 antibody, and (C) both antibodies, showing that Rab11 and Rip11DN-GFP co-localize in punctate vesicles. (D-F) 1096GAL4>Rip11DN+EF salivary glands stained with a rabbit anti-GFP antibody (D), a mouse anti-Rab11 antibody (E), and both antibodies (F), showing that Rab11 and Rip11DN still co-localize in EF-expressing glands. However, EF alters the distribution of both proteins, transforming small punctate staining into a ring-shaped halo surrounding secretory vesicles.
(TIF)

S8 Fig ET treatment reduces Rab11/Rip11 co-localization in MDCK cells. Co-localization between Rip11-GFP and DsRed-Rab11A in co-transfected MDCK cells measured by the Pearson's correlation coefficient (PCC) is reduced by ET treatment (n = 43, p<4.85X10^-9^).
(TIF)

S9 Fig ET treatment reduces Sec15/Rab11* and Rab11*/Rip11 co-localization in HBMEC cells. (A-C) HBMECs, untreated. (D-F) HBMECs treated with ET for 6hours. Co-localization of Rab11* with Sec15 (B and E) and Rab11* with Rip11 (C and F) can be visualized following transfection of cells with Sec15-GFP. High-level expression of Sec15-GFP, and staining with an anti-Rab11* antibody (A) reveals a high degree of Sec15/Rab11* co-localization (B). In ET-treated cells, this co-localization is severely reduced (E). A double label Rab11*/Rip11 stain, reveals Rab11*/Rip11 co-localization (C), which is also abrogated by ET (F).
(TIF)

S10 Fig Arf6RNAi rescues normal apical D-Ecad levels in EF-expressing wing discs. Apical levels of D-Ecad in wing discs was measured using ImageJ. Arf6RNAi restores normal levels of apical D-Ecad in 1096GAL4>EF+Arf6RNAi discs (p<0.0001). Arf6RNAi does not notably affect apical levels of D-ECad. Surface areas of wings of the same genotypes were also measured, and Arf6RNAi showed a modest yet significant restorative effect in EF-expressing wings (in 1096GAL4>EF+Arf6RNAi wings, p<0.05).
(TIF)

S11 Fig ET treatment reduces the levels of cadherins and Rab11 in HBMECs. (A-B) Western blot analysis of HBMECs cells. (A) Rab11A (~28 kD) and pan-Cadherin (~97 kD) levels are severely decreased in ET-treated cells (24hrs), while control actin (~42 kD) levels are only slightly reduced. (B) dcAMP, a cell-permeant stable analog of cAMP that is insensitive to phosphodiesterase, also causes a severe loss of Rab11, suggesting that the reduction of Rab11 levels induced by ET is mediated by cAMP.
(TIF)
