# Supplementary figures and images for: Anthrax edema toxin disrupts distinct steps in Rab11-dependent junctional transport
Source: PLoS Pathog. 2017 Sep 25;13(9):e1006603. doi: 10.1371/journal.ppat.1006603 (PMC5612732; doi:10.1371/journal.ppat.1006603)

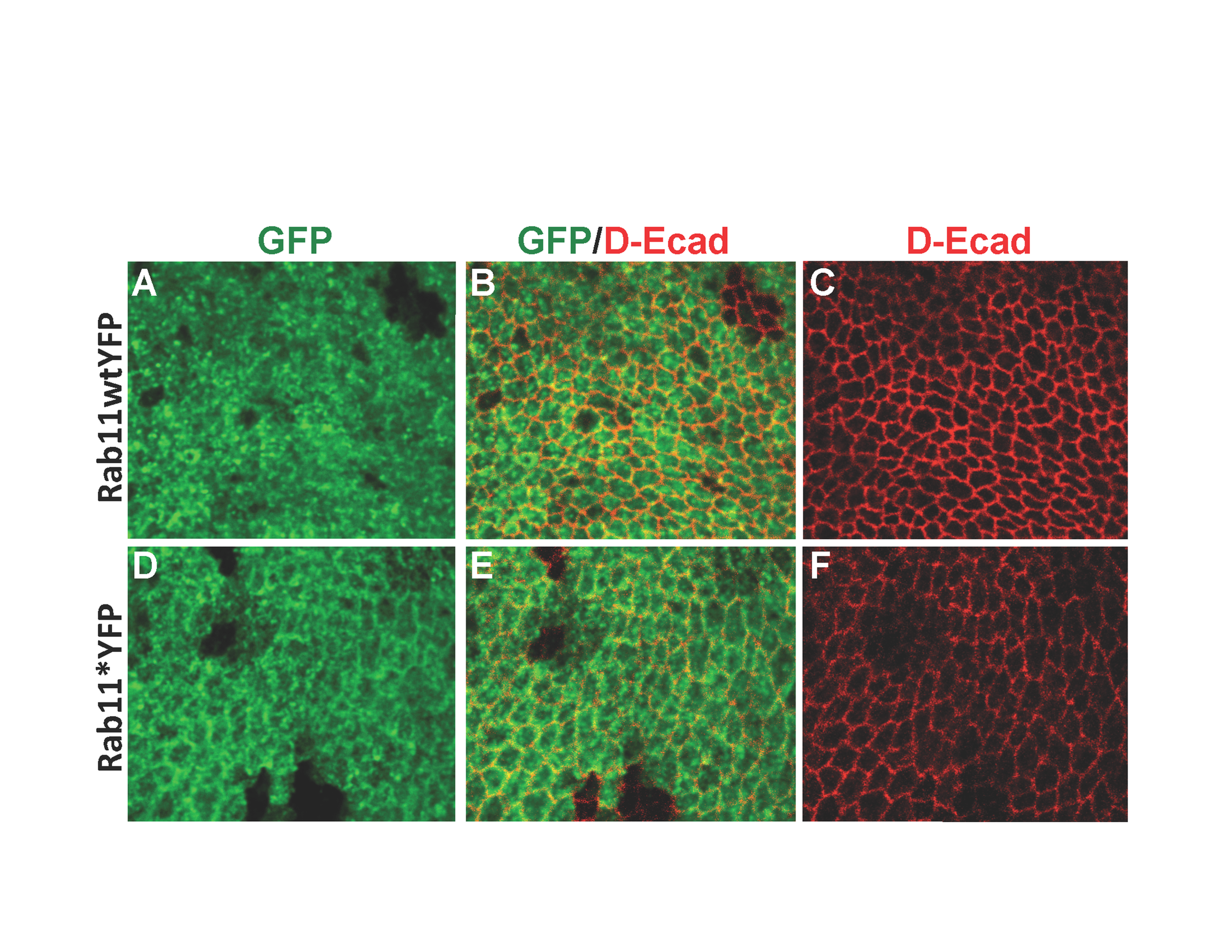

Supplement: S1 Fig — (A-C) Expression of Rab11wt in 1096GAL4>Rab11wtYFP wing discs. (A) Rab11wtYFP detected with a rabbit anti-GFP antibody appears as a peppered stain near the apical surface. (B) D-Ecad/GFP double stain. (C) corresponding D-Ecad stain marking AJs. (D-F) Expression of Rab11* in 1096GAL4>Rab11*YFP wing discs. (D) Rab11*YFP detected with a rabbit anti-GFP antibody. In addition to a peppered apical stain, Rab11* shows a distinctive net-like pattern at cell borders. (E) D-Ecad/GFP double stain, revealing that Rab11*YFP tends to accumulate at the AJs. (F) Corresponding D-Ecad single stain. (TIF) [file ppat.1006603.s001.tif]

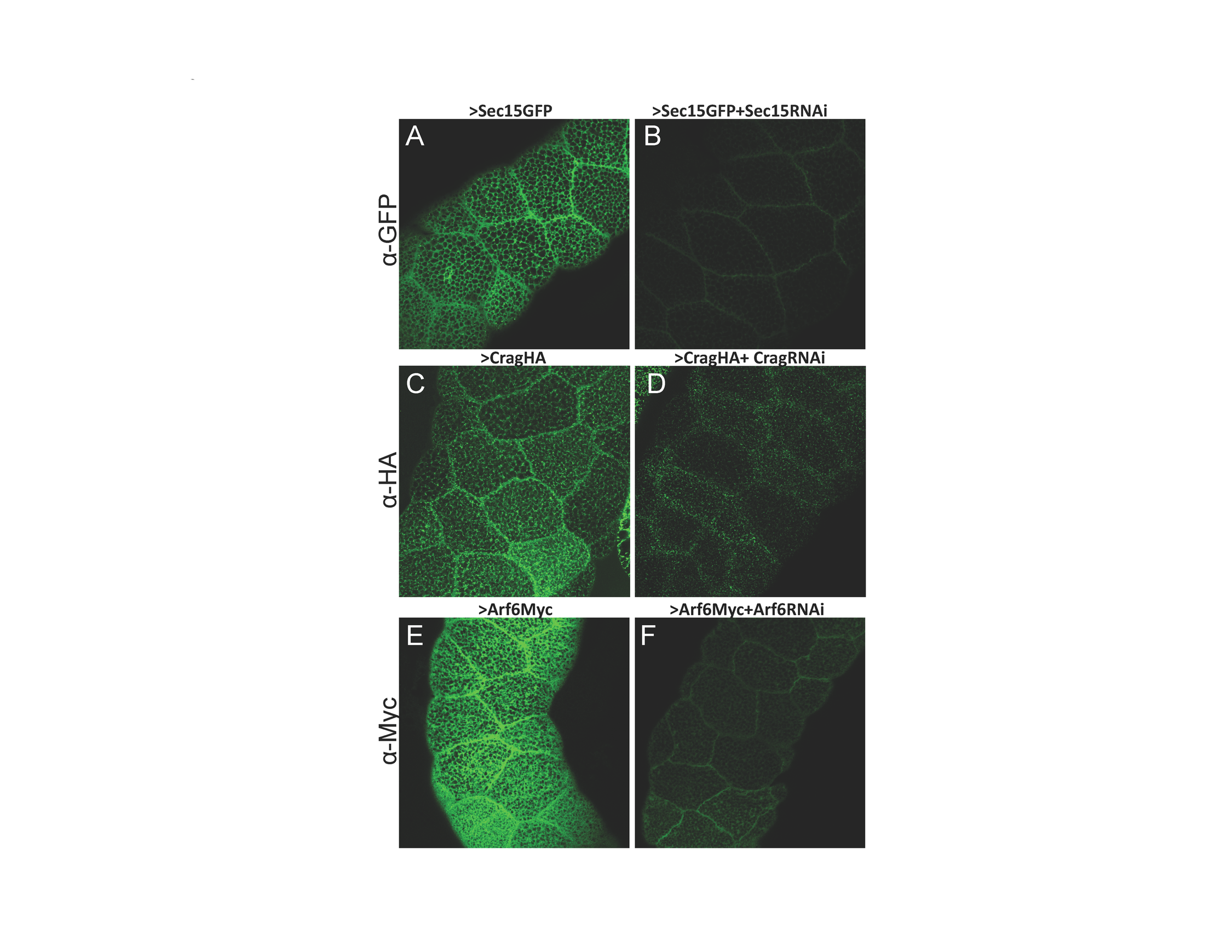

Supplement: S2 Fig — (A) A 1096GAL4>UAS-Sec15GFP salivary gland, stained with an anti-GFP antibody. (B) Sec15GFP expression in strongly inhibited by co-expression of a Sec15RNAi construct. (C) A 1096GAL4>UAS-CragHA salivary gland stained with an anti-HA antibody. (D) CragHA expression is suppressed by co-expression of a CragRNAi construct. (E) A 1096GAL4>UAS-Arf6Myc gland stained with an anti-Myc antibody. (F) Arf6Myc expression is blocked by co-expression of an Arf6RNAi construct. (TIF) [file ppat.1006603.s002.tif]

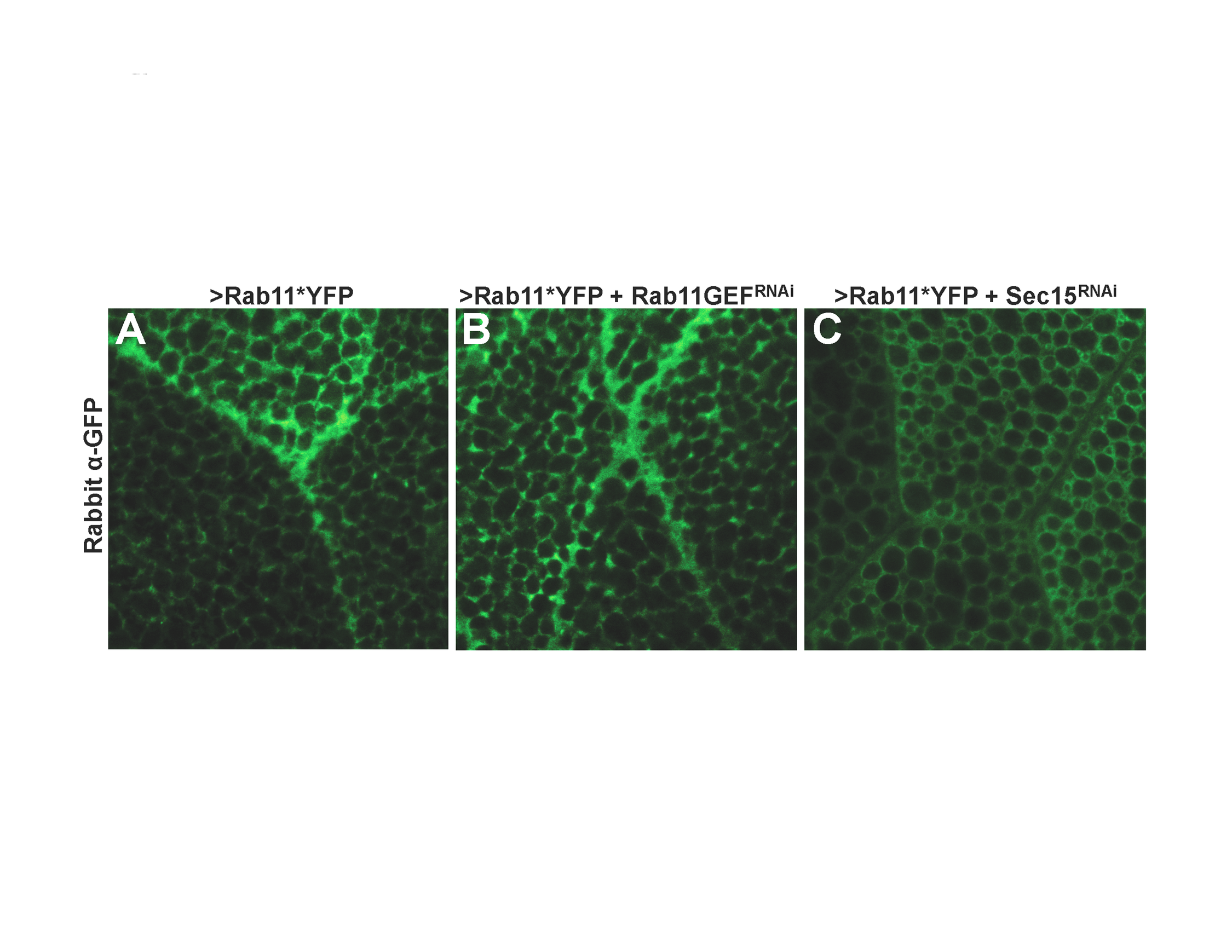

Supplement: S3 Fig — (A-C) Rab11*YFP detected with a rabbit anti-GFP antibody in salivary glands. (A) Rab11*YFP selectively accumulates at the AJs in 1096GAL4>Rab11*YFP salivary glands. (B) Rab11* distribution is unchanged in 1096GAL4>Rab11*YFP+CragRNAi glands. (C) Rab11*YFP fails to accumulate at the AJs in 1096GAL4>Rab11*YFP +Sec15RNAi salivary glands. (TIF) [file ppat.1006603.s003.tif]

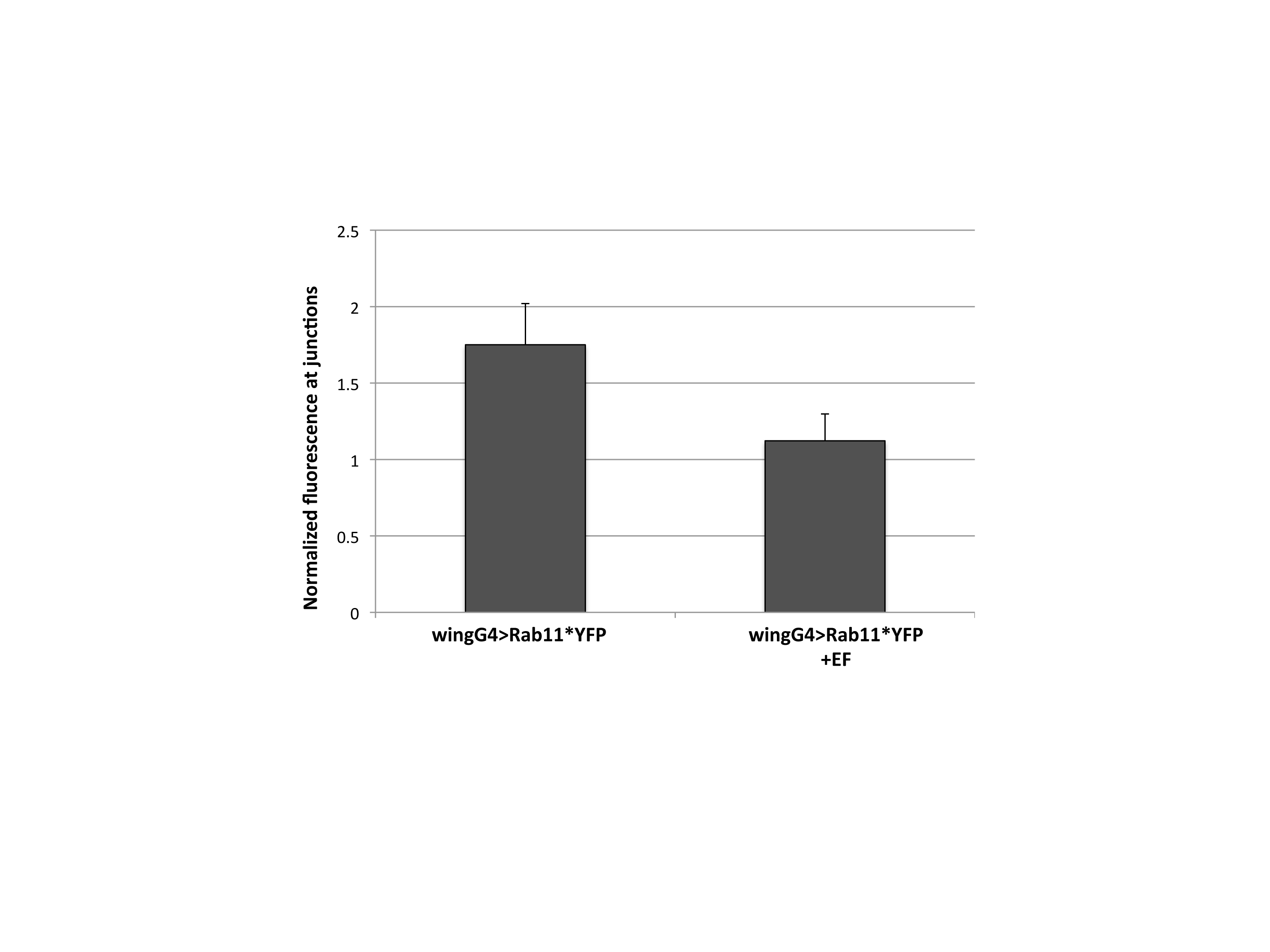

Supplement: S4 Fig — Images from experiment described in Fig 1, panels E, F, H and I, were analyzed to quantify the effect of EF on junctional accumulation of Rab11*. Individual image crops from intercellular boundaries were generated. For each crop, average fluorescence was determined in ImageJ, and normalized to the average fluorescence found inside the corresponding cell. EF expression significantly reduces Rab11* accumulation at intercellular borders, (p<0.0001). (TIF) [file ppat.1006603.s004.tif]

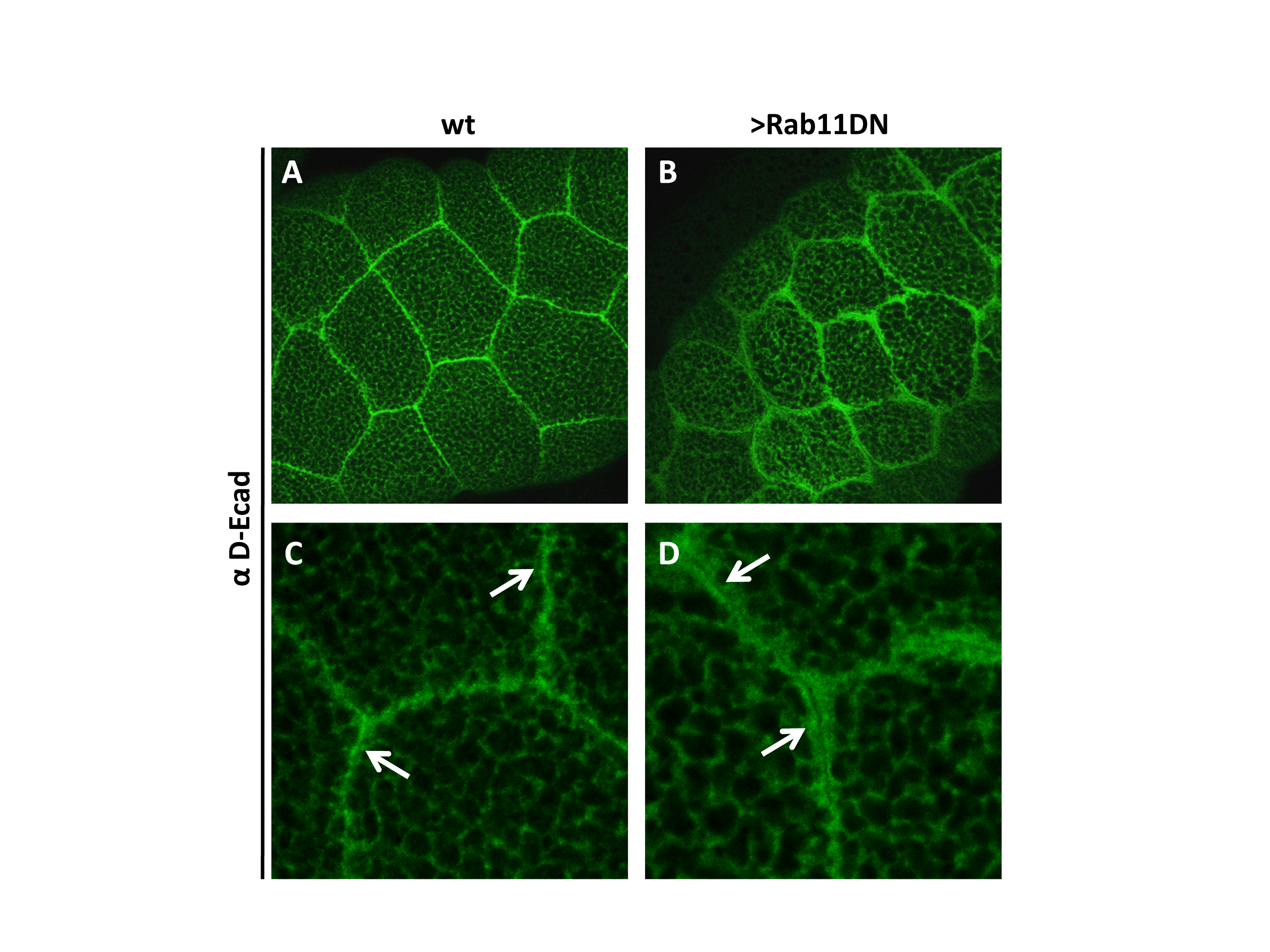

Supplement: S5 Fig — (A-D) Salivary glands stained with an anti-D-Ecad antibody. (A) A wild-type salivary gland showing D-Ecad accumulation at AJs. (B) A SglGAL4>Rab11DN salivary gland, in which Rab11 inhibition in this tissue leads to D-Ecad accumulation in broad zones around intercellular gaps. (C-D) Higher magnifications. (C) A wild-type salivary gland showing D-Ecad forming AJs (arrows). (D) A SglGAL4>Rab11DN salivary gland, revealing gaps between cells, and broad accumulation of D-Ecad around them (arrows). D-Ecad fails to form AJs. (TIF) [file ppat.1006603.s005.tif]

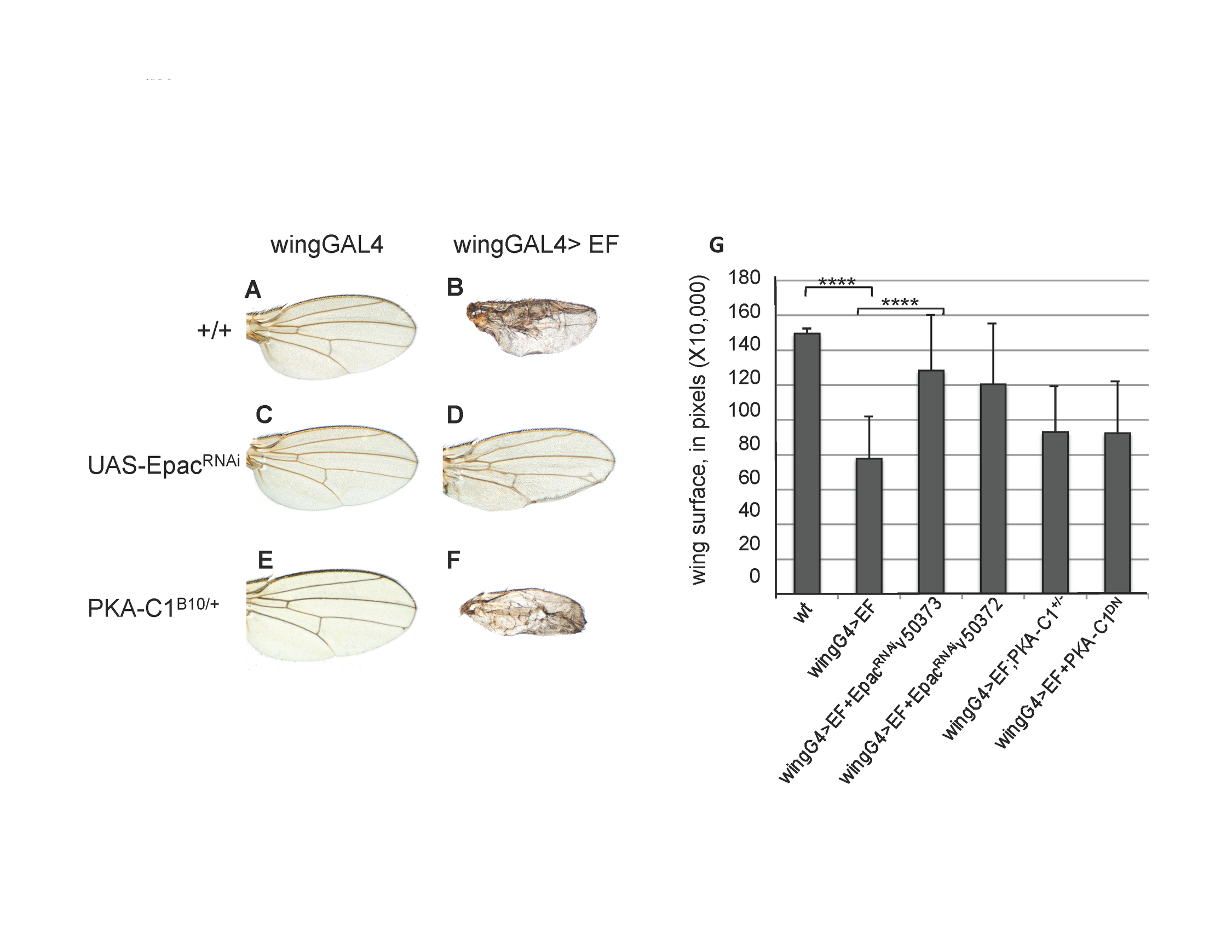

Supplement: S6 Fig — (A-F) wings of the following genotypes: (A) Wild-type (+/+). (B) 1096GAL4>EF. (C) 1096GAL4>EpacRNAi. (D) 1096GAL4>EF+EpacRNAi. Inhibition of Epac expression potentlyreduces the EF phenotype. (E) PKA-C1B10/+ (B10 is a loss -of-function allele of PKA). (F) 1096GAL4>EF; PKA-C1B10/+. Reduction of PKA-C1 levels, either in a heterozygote loss-of-function PKA-C1 alleles (B10/+) or in flies expressing a dominant negative form of PKA-C (C1-DN), does not obviously alter the EF phenotype. (G) The surface areas of wings of the indicated genotypes were measured in Photoshop. Results were plotted as a histogram, with relevant p-values indicated. EF expression reduces wing size significantly compared to widl-type (wt) (****p<0.0001). EpacRNAi ameliorates the EF phenotype (****p<0.0001). (TIF) [file ppat.1006603.s006.tif]

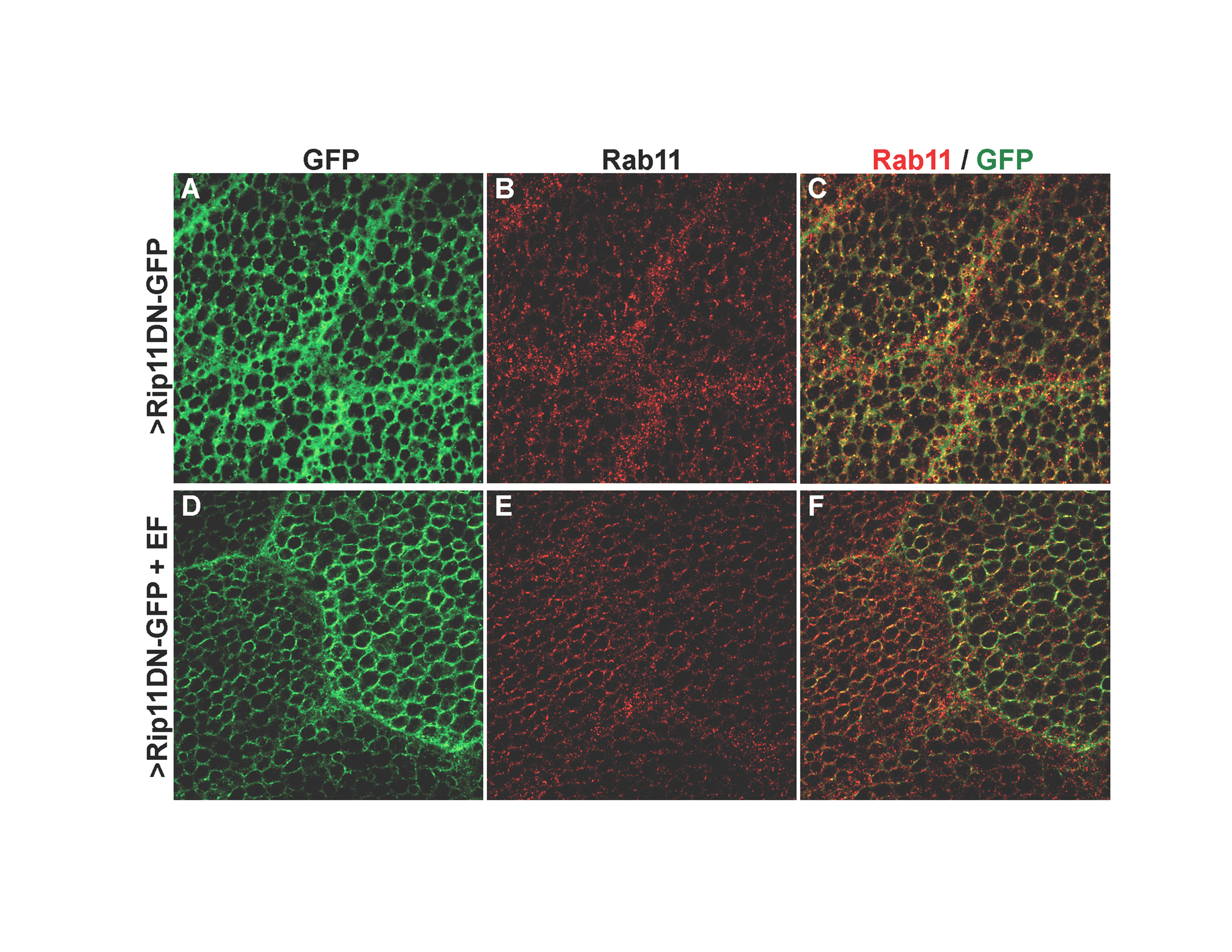

Supplement: S7 Fig — (A-C) 1096GAL4>Rip11DN-GFP salivary glands, stained with (A) a rabbit anti GFP antibody, (B) a mouse anti Rab11 antibody, and (C) both antibodies, showing that Rab11 and Rip11DN-GFP co-localize in punctate vesicles. (D-F) 1096GAL4>Rip11DN+EF salivary glands stained with a rabbit anti-GFP antibody (D), a mouse anti-Rab11 antibody (E), and both antibodies (F), showing that Rab11 and Rip11DN still co-localize in EF-expressing glands. However, EF alters the distribution of both proteins, transforming small punctate staining into a ring-shaped halo surrounding secretory vesicles. (TIF) [file ppat.1006603.s007.tif]

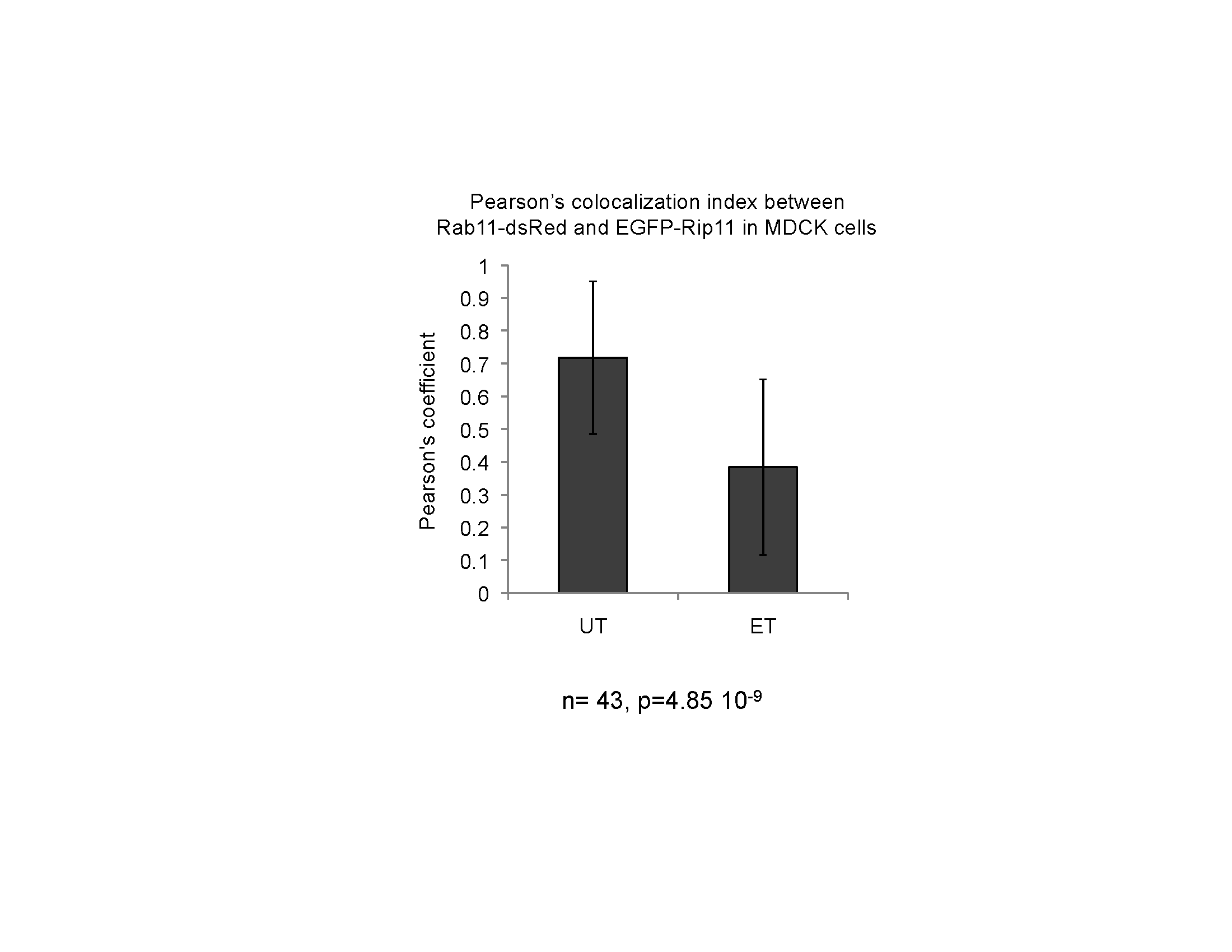

Supplement: S8 Fig — Co-localization between Rip11-GFP and DsRed-Rab11A in co-transfected MDCK cells measured by the Pearson's correlation coefficient (PCC) is reduced by ET treatment (n = 43, p<4.85X10-9). (TIF) [file ppat.1006603.s008.tif]

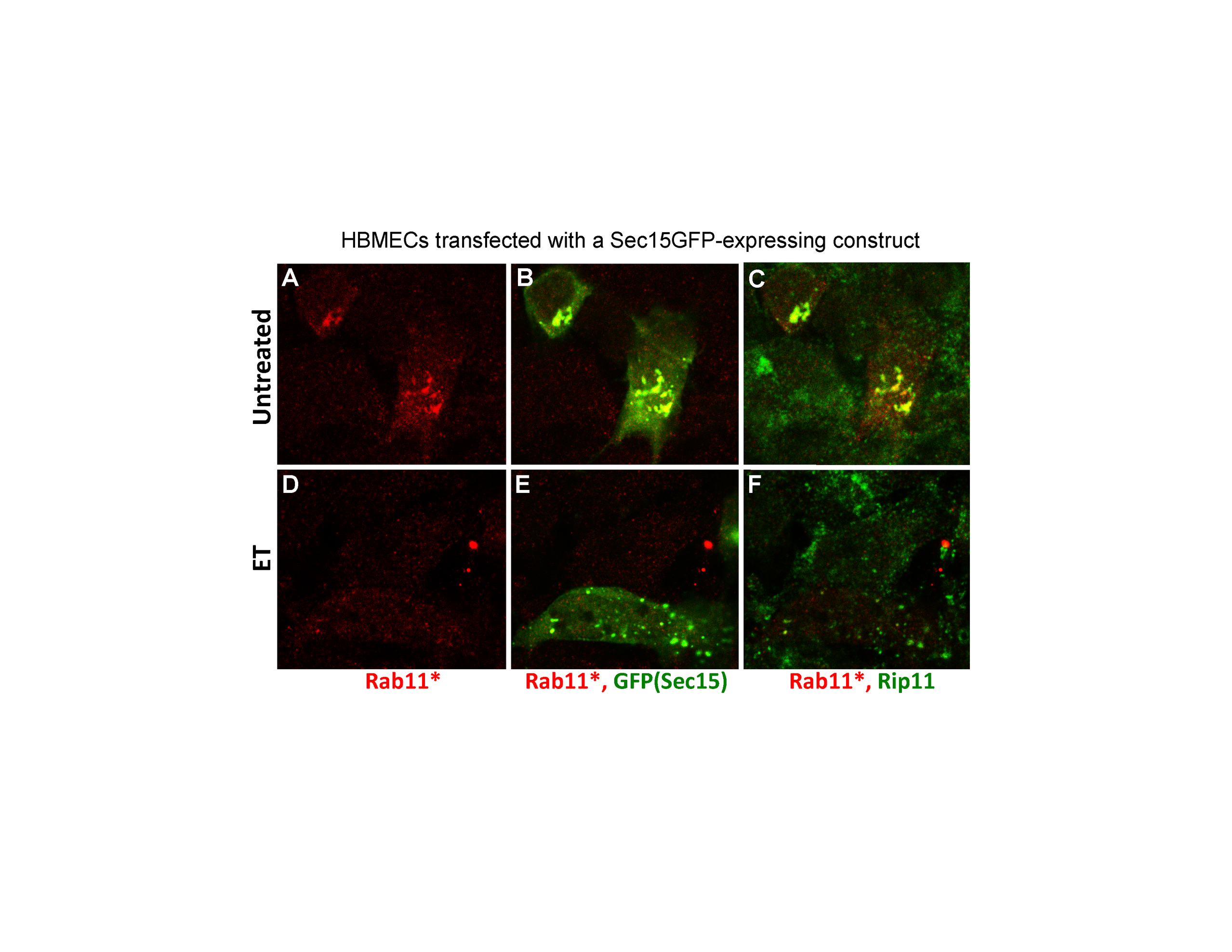

Supplement: S9 Fig — (A-C) HBMECs, untreated. (D-F) HBMECs treated with ET for 6hours. Co-localization of Rab11* with Sec15 (B and E) and Rab11* with Rip11 (C and F) can be visualized following transfection of cells with Sec15-GFP. High-level expression of Sec15-GFP, and staining with an anti-Rab11* antibody (A) reveals a high degree of Sec15/Rab11* co-localization (B). In ET-treated cells, this co-localization is severely reduced (E). A double label Rab11*/Rip11 stain, reveals Rab11*/Rip11 co-localization (C), which is also abrogated by ET (F). (TIF) [file ppat.1006603.s009.tif]

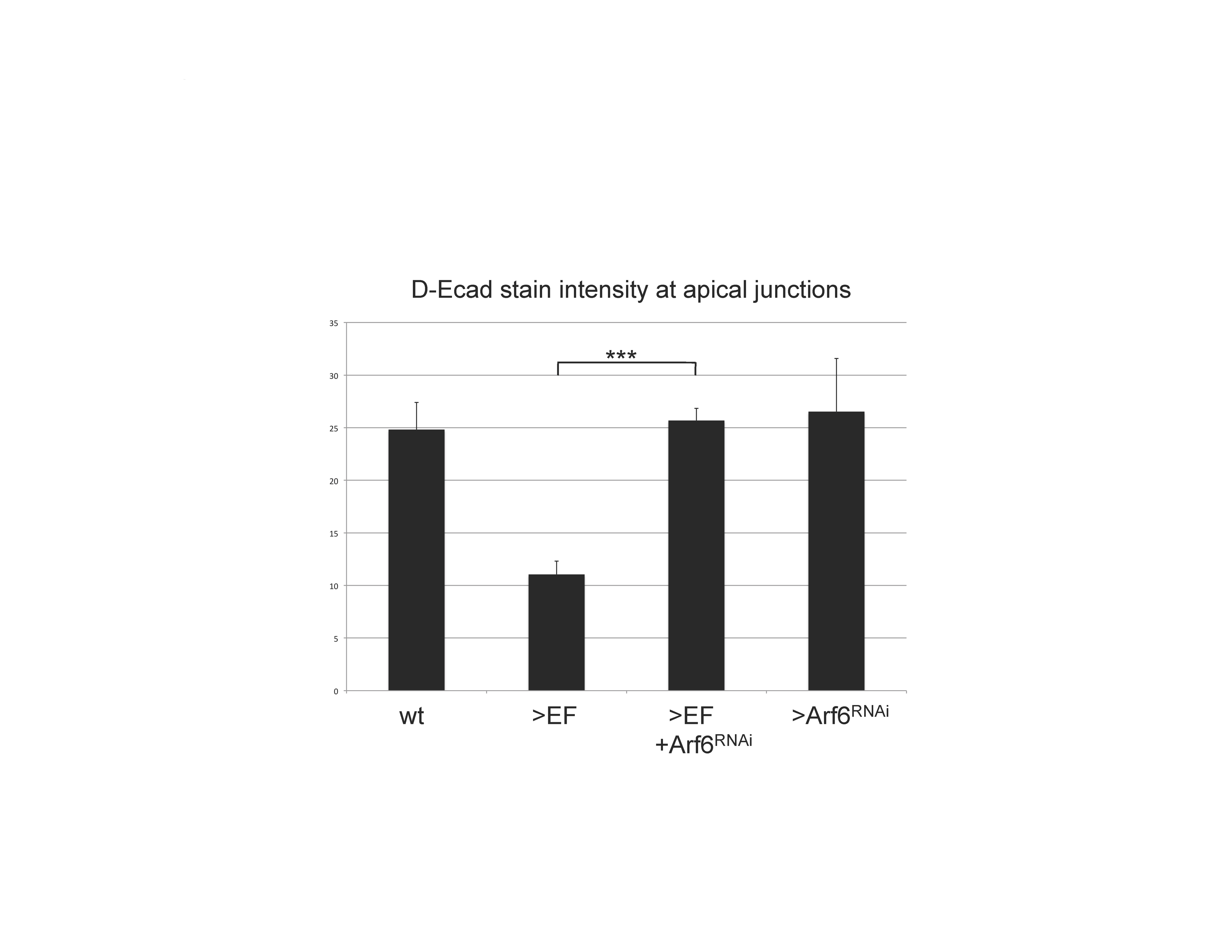

Supplement: S10 Fig — Apical levels of D-Ecad in wing discs was measured using ImageJ. Arf6RNAi restores normal levels of apical D-Ecad in 1096GAL4>EF+Arf6RNAi discs (p<0.0001). Arf6RNAi does not notably affect apical levels of D-ECad. Surface areas of wings of the same genotypes were also measured, and Arf6RNAi showed a modest yet significant restorative effect in EF-expressing wings (in 1096GAL4>EF+Arf6RNAi wings, p<0.05). (TIF) [file ppat.1006603.s010.tif]

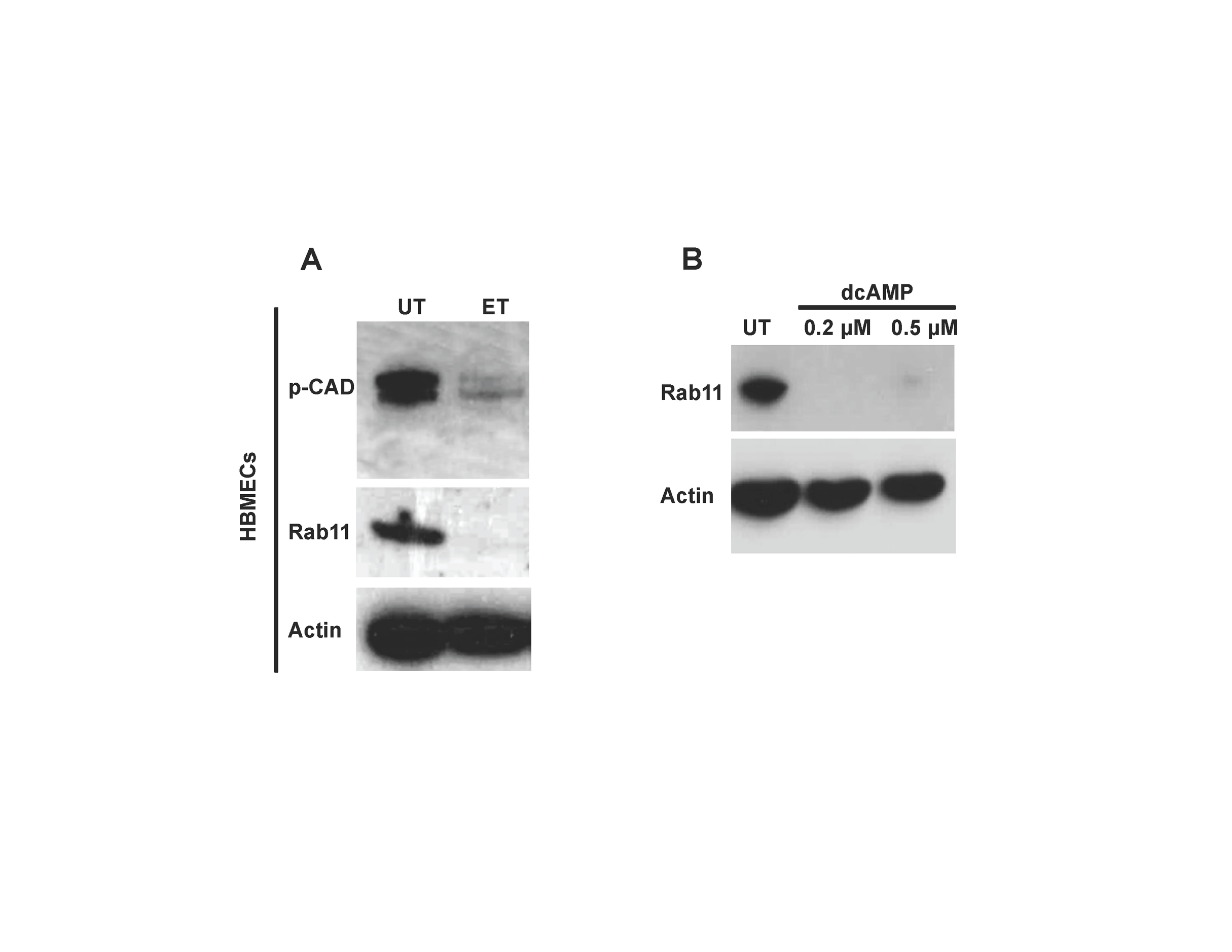

Supplement: S11 Fig — (A-B) Western blot analysis of HBMECs cells. (A) Rab11A (~28 kD) and pan-Cadherin (~97 kD) levels are severely decreased in ET-treated cells (24hrs), while control actin (~42 kD) levels are only slightly reduced. (B) dcAMP, a cell-permeant stable analog of cAMP that is insensitive to phosphodiesterase, also causes a severe loss of Rab11, suggesting that the reduction of Rab11 levels induced by ET is mediated by cAMP. (TIF) [file ppat.1006603.s011.tif]
